# Genome‐wide profiling of N6‐methyladenosine‐modified pseudogene‐derived long noncoding RNAs reveals the tumour‐promoting and innate immune‐restraining function of RPS15AP12 in ovarian cancer

**DOI:** 10.1002/ctm2.70249

**Published:** 2025-02-25

**Authors:** Jie Xu, Yifei Ren, Jiayi Lu, Fengjiang Qin, Dan Yang, Chunyan Tang, Yu Yang, Jing Xu, Tao Liu, Ping Yi

**Affiliations:** ^1^ Department of Obstetrics and Gynecology The Third Affiliated Hospital of Chongqing Medical University Chongqing China; ^2^ Department of Obstetrics and Gynecology Daping Hospital Army Medical University Chongqing China; ^3^ Department of Obstetrics and Gynecology Chongqing University Fuling Hospital Chongqing China; ^4^ Department of Obstetrics and Gynecology Women and Children's Hospital of Chongqing Medical University Chongqing China

**Keywords:** innate immune response, m^6^A, miRNA sponge, ovarian cancer, pseudogene

## Abstract

**Background:**

Pseudogene‐derived lncRNAs are widely dysregulated in cancer. Technological advancements have facilitated the functional characterization of increasing pseudogenes in cancer progression. However, the association between pseudogenes and RNA N6‐methyladenosine (m^6^A) modification in cancer, as well as the underlying mechanisms, remains largely unexplored.

**Methods:**

We analyzed the expression of 12 146 pseudogenes and comprehensively examined the m^6^A modification of RNAs derived from them and their paralogs. Through integrative analysis of multi‐omics data, we explored the associations between pseudogene dysregulation and m^6^A, identifying critical pseudogenes involved in HGSOC progression. Tumour promotion role of RPS15AP12 and its cognate parent gene was characterized by cell proliferation, transwell assays, and scratch assays in ovarian cells and xenograft nude mice. RNA decay assays were used to reveal the participation of m^6^A in decreasement of RPS15AP12 lncRNA stability. Luciferase reporter assays were performed to verify that RPS15AP12 enhances RPS15A expression by competitively binding to miR‐96‐3p. Western blot and phosphorylation assays were performed to investigate the impairment of RPS15AP12 towards the sensors of MAVS (RIG‐I and MDA5), and downstream p‐TBK1 and p‐IRF3. Finally, ELISA assays were performed to explore the regulatory role of RPS15AP12 in IFN‐β expression.

**Results:**

M^6^A is distributed across over a thousand pseudogenes, and hypomethylation leads to their upregulation in HGSOC. We identified a processed pseudogene, RPS15AP12, upregulated by FTO‐mediated m^6^A demethylation. RPS15AP12 enhances the growth ability and metastatic capabilities of ovarian cancer (OC) cells via functioning as a competitive endogenous RNA (ceRNA) for its host gene, RPS15A, through the sequestration of miR‐96‐3p. Importantly, the deletion of RPS15AP12 diminishes the expression of RPS15A, leading to the upregulation of anti‐tumour immune responses by activating RIG‐I and MDA5 and downstream p‐TBK1 and p‐IRF3 as well as IFN‐β levels.

**Conclusion:**

Our findings expand the understanding of m^6^A‐modulated pseudogenes in tumour growth and anti‐tumour innate immunity in OC.

**Key Points:**

- Genome‐wide profiling reveals the redistribution of m6A modification on pseudogene‐derived lncRNAs and m6A redistribution‐relevant dysregulation of pseudogenes in HGSOC.
- RPS15AP12, as a representative processed pseudogene, is up‐regulated by FTO‐mediated demethylation and acts as a miRNA sponge to promote RPS15A expression via competitively binding to miR‐96‐3p.
- RPS15AP12/RPS15A axis inhibits MAVS sensors (RIG‐I and MDA5) and downstream IFN‐β levels in ovarian cancer.

## Graphical abstract

Genome‐wide profiling reveals the redistribution of m6A modification on pseudogene‐derived lncRNAs and m6A redistribution‐relevant dysregulation of pseudogenes in HGSOC. RPS15AP12, as a representative processed pseudogene, is up‐regulated by FTO‐mediated demethylation and acts as a miRNA sponge to promote RPS15A expression via competitively binding to miR‐96‐3p. The RPS15AP12/ RPS15A axis exhibits an anti‐tumour effect via inhibiting innate immune response in ovarian cancer.

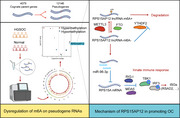

## 1 BACKGROUND

Pseudogenes, once considered non‐functional genomic relics of evolution,^1^ are generated through mutations that disable their protein‐coding ability. Advances in technology, such as CRISPR‐Cas9 and whole‐genome sequencing, have facilitated the functional analysis and expression profiling of pseudogenes, challenging the notion that they lack function. Pseudogenes are primarily transcribed as long noncoding RNAs (lncRNAs),^2^, ^3^ and their transcription levels have been identified as tumour subtype‐specific in a pan‐cancer analysis, underscoring their clinical relevance.^4^ They play a significant role in innate immunity‐related pathways. Previous research has highlighted pseudogenes’ roles in antiviral immunity, including RNA5SP141, Lethe, and RNVU1‐18.^5^, ^6^, ^7^ Additionally, pseudogenes are involved in immune‐related anti‐tumour effects; for example, SIRPAP1 affects the efficacy of anti‐PD‐L1 treatment in melanoma through ceRNA regulation of its paralog SIRPA.^8^ BRCA1P1 lncRNA binds to the NF‐κB subunit RelA, inhibiting its activation and interferon‐stimulated genes (ISGs) expression, thus demonstrating an oncogenic role.^9^ However, despite the progress in next‐generation sequencing, a gap remains between the number of annotated pseudogenes and those functionally characterized, leading to calls for a re‐evaluation of the functions of these annotated pseudogenes.^10^

Recent genetic research has revealed that processed pseudogenes have accumulated de novo m^6^A sites under positive natural selection.^11^ m^6^A, the most prevalent modification in mRNAs, also regulates non‐coding RNAs (ncRNAs), including cleavage, localization, transport, stability, and degradation.^12^, ^13^, ^14^, ^15^, ^16^ Pseudogenes, due to their accumulated nonsense mutations, are primary targets of nonsense‐mediated mRNA decay (NMD).^11^ However, processed pseudogenes, constituting 72% of pseudogenes and generated through retro‐transposition of mRNA transcripts, lack introns and are thus not typical NMD substrates. Consequently, the regulation by m^6^A of pseudogenes is crucial, yet its implications in oncogenesis remain poorly understood, necessitating further exploratory studies.

OC has the highest lethality among gynaecological malignancies,^17^ and significant improvement in overall survival (OS) has not been achieved in the past few decades.^18^ The disease often begins asymptomatically and lacks effective screening methods, resulting in over 75% of patients being diagnosed at an advanced stage with a 5‐year survival rate of 29%, compared with 92% for those diagnosed at an early stage. HGSOC comprises 70% of all OC cases and nearly 80% of the associated deaths.^19^ Research into molecular and functional profiling of HGSOC seeks to identify novel therapeutic and diagnostic targets. Notably, several pseudogene‐derived lncRNAs, including HMGA1P6, CTSLP8, and SDHAP1, have been implicated in the progression of OC.^20^, ^21^, ^22^ However, the role of m^6^A‐mediated RNA surveillance in OC remains unexplored and merits further research.

In this study, the redistribution of m^6^A modification on pseudogene‐derived lncRNAs and associated dysregulation of pseudogenes in HGSOC were observed. Pseudogenes, including RPS15AP12 which is upregulated by FTO‐mediated demethylation, were identified as potential targets in OC. RPS15AP12, the twelfth pseudogene of the highly conserved 40S ribosomal protein S15A (RPS15A), is involved in the mRNA/ribosome interactions during early translation.^23^ In cancer, RPS15A is reported to activate NF‐κB/AKT, p53, and Wnt/β‐catenin signalling pathways^23^, ^24^, ^25^; however, the mechanisms remain poorly understood. The current study reveals that RPS15AP12 enhances RPS15A expression by competitively binding to miR‐96‐3p and inhibiting MAVS sensors (RIG‐I and MDA5) and downstream IFN‐β levels. These findings expand our understanding of m^6^A‐modulated pseudogenes in tumour growth and anti‐tumour innate immunity in OC.

## 2 METHODS AND MATERIALS

### 2.1 Public m^6^A RNA immunoprecipitation data

We used the Sequence Read Archive (SRA) database to obtain publicly available m^6^A RNA immunoprecipitation (MeRIP)‐seq data from clinical samples of project PRJNA488293 (https://trace.ncbi.nlm.nih.gov/Traces/index.html?view=run_browser&acc=SRR7763558&display=metadata). Prior informed consent was obtained for the collection of all human tissue specimens in accordance with the Declaration of Helsinki and the University of Chicago's Institutional Review Board's ethical standards. The approved protocols were followed in the experiments. During primary debulking procedures performed at the University of Chicago, six omental tumour samples were collected from patients with recently identified advanced, metastatic HGSOC. Patients undergoing surgical procedures for benign gynaecological disorders yielded seven normal fallopian tube specimens.^26^

### 2.2 Clinical tissue samples

Patients undergoing OC at the Third Affiliated Hospital of Chongqing Medical University provided normal fallopian tube epithelial tissues and OC tissues. Skilled pathologists confirmed the results after carefully analyzing the material. These patients were just starting on their treatment regimen and had not yet had any kind of surgery or chemotherapy. Every participant gave their informed consent after the study was approved by the Accreditation Committee of the Third Affiliated Hospital of Chongqing Medical University.

### 2.3 Sample processing of ovarian cell line RNA‐seq

Trizol reagent (Invitrogen) was used to isolate total RNA from OC cells following recognized methods. The PolyATtract mRNA Isolation System (Promega) was subsequently used to separate the messenger RNA (mRNA) from the total RNA. New England BioLabs's NEB Next Ultra RNA Library Prep Kit for Illumina was used to build RNA libraries, and the Illumina NovaSeq 6000 Sequencing System generated an average of about 50 million pairs of paired‐end reads, each of which was 150 nucleotides long.

### 2.4 Analysis of the RNA‐seq data

The Illumina HiSeq X Ten platform was used for the sequencing, and the paired‐end read length was 150 bp. Using the default settings of TopHat2 (v2.1.1), all reads were mapped to the Homo sapiens genome hg38. Reads that were perfectly matched were the only ones included; for downstream analysis, only reads that were properly paired and uniquely mapped were chosen.^11^ Utilizing HTSeq (v2.2.1), the number of reads was measured. The differentially expressed genes’ log2 fold changes were determined with the help of DESeq2 (version 1.26.0).

### 2.5 Analysis of the MeRIP‐seq data

Aligning clear reads from HGSOC samples to the Homo sapiens genome hg38 was done using Hisat2 v2.1.1.^27^ Using the R package exomePeak2 with the default settings, we detected m^6^A‐enriched peaks in each m^6^A ‐IP sample, using the corresponding input sample as a control.^28^ Using exomePeak2 (version 3.7),^28^ we compared the m^6^A peaks of metastatic and normal samples. Peaks with *p*‐value < .05 were considered significant and annotated by the annotatePeaks function of HOMER.^29^ To facilitate genomic visualization, the mapping reads were transformed into normalized BigWig files using Deeptools (version 3.4.1).^30^ These files were then normalized using CPM, with read coverage for each 100 bp window calculated as the number of mapped reads per million. To see the m^6^A peaks, the Integrative Genomics Viewer (IGV) tool was used. For the integrative analysis of relative m^6^A modification level (tristile) and gene expression, the background transcriptome level was the count of reads in each m^6^A‐input sample.

### 2.6 Identification of enriched motifs within m^6^A peaks

The motifs that were found to be more prevalent in the target m^6^A peak sequences, as opposed to the control sequences (shuffled m^6^A peak sequences), were found to be short (≤8 bp) and ungapped using the Discriminative Regular Expression Motif Elicitation (DREME) suite of MEME.^31^ Using the fastaFromBed tool in BEDTools software v2.28,^32^ the target m^6^A peak sequences were retrieved from the human reference genome hg38. While maintaining the nucleotide frequencies, the control sequences were created by randomly shuffling each m^6^A peak sequence.

### 2.7 Cell lines and cell culture

The American Type Culture Collection provided the HEK293T cells, while the Cell Bank of the Chinese Academy of Medical Sciences supplied the OVCAR3 and SKOV3 cell lines. OVCAR3 cells were grown in RPMI 1640 media (GIBCO), HEK293T and SKOV3 cells in DMEM (GIBCO), with 10% fetal bovine serum (GIBCO) added. The cells were cultivated at 37°C in a 5% CO_2_ environment. Before conducting experiments, it was ensured that all cell lines were free of mycoplasma contamination.

### 2.8 Plasmids, cell transfection, and lentiviral infection

The pLKO.1 vector (Addgene) with puromycin selection was used to clone the short hairpin RNAs (shRNAs) targeting METTL3, FTO, YTHDF2, and RPS15A (sh1, sh2), as well as the negative control (shNC). In the supplemental table, you may find the sequences of the shRNAs. A plasmid called pCDH‐PURO was used to clone full‐length RPS15AP12 (Addgene). The pcDNA3.1 vector (Youbio) was used to overexpress RPS15AP12 and RPS15A cDNAs. Sangon Biotech of Shanghai sold a plasmid extraction kit. Using jetPRIME (Polyplus) as directed by the manufacturer, plasmid transfections were carried out. After 48 h of transfection, the quantities of messenger RNA and protein were measured. Utilizing jetPRIME transfection (Polyplus), lentiviral vectors were delivered into HEK293T cells alongside packaging vectors psPAX2 (#12260, Addgene) and pMD2.G (#12259, Addgene). After 48 h of transfection, the lentiviruses were collected.

### 2.9 Establishment of RPS15AP12 knockout (KO) cell lines

Through the use of CRISPR/Cas9 technology, RPS15AP12 KO cell lines were produced. To summarize, the LvSG06‐RP15AP12‐KO plasmid was constructed by cloning sgRNA#1 (GTCTTTCTGCACCACCACAA) and sgRNA#2 (TTTACAAATAAAATGCCCCG) targeting RPS15AP12 into the pCRISPR‐LvSG06‐puro (GeneCopoeia). To achieve RPS15AP12 KO in OVCAR3 and SKOV3 cells, cells in 6 cm pans were transfected with 2 µg of the LvSG06‐RP15AP12‐KO plasmid using jetPRIME (Polyplus) at a density of 60–70%. The cells were chosen using 2 µg/mL puromycin 48 h after transfection.

### 2.10 RNA extraction and quantitative real‐time PCR

To extract total RNA, we used the manufacturer's instructions for Trizol (Sigma). Using Vazyme's HiScript II Q RT SuperMix for qPCR, cDNA was generated from whole RNA. We used a QuantStudioDx device from Life Technologies to perform quantitative real‐time PCR (RT‐qPCR) with SYBR Green Master Mix from Vazyme. The relative expression level of the gene was calculated using the 2^−△△Ct^ technique. In the supplementary table, the primers for RT‐qPCR were purchased from Tsingke Biotech in Beijing.

### 2.11 Western blot

A BCA detection kit (Solarbio) was used to quantify protein contents after lysing cell pellets with RIPA buffer (Beyotime) that included 1% PMSF. Membranes made of PVDF (Millipore) were used for protein transfer after 10–12% SDS‐PAGE separation. After 1 h of blocking with 5% skim milk, the membranes were incubated with primary antibodies at 4°C overnight. Primary antibodies were as follows: METTL3 (1:1000, 15073‐1‐AP, Proteintech), FTO (1:1000, 27226‐1‐AP, Proteintech), YTHDF2 (1:2000, 24744‐1‐AP, Proteintech), RPS15A (1:1000, TA369533S, ORIGENE), MDA5 (1:2000, 21775‐1‐AP, Proteintech), RIG‐1 (1:1000, 20566‐1‐AP, Proteintech), TBK1 (1:1000, #3504, CST), p‐TBK1 (1:1000, 82383‐1‐RR, Proteintech), IRF3 (1:5000, 11312‐1‐AP, Proteintech), p‐IRF3 (1:1000, 29528‐1‐AP, Proteintech), GAPDH (1:10000, 60004‐1‐Ig, Proteintech). Membranes were incubated with the secondary antibody for 1 h at room temperature after being washed three times with TBST. Chemiluminescence on a western blot Imaging System was used to visualize the positive bands.

### 2.12 Enzyme‐linked immunosorbent assays

Growth medium supernatants were collected from the cell samples and subjected to IFN‐β measurement by enzyme‐linked immunosorbent assays (Human IFN‐beta RUNXIN, R&D Systems). The protein concentration of cell samples at harvesting was measured and used to normalize IFN‐β read‐outs.

### 2.13 Immunohistochemistry

Prior to heat‐mediated antigen retrieval, paraffin‐embedded tissue sections underwent deparaffinization. Incubation of the slides with primary antibodies against Ki‐67 (AF0198, Affinity, 1:100), Caspase‐3 (9662S, CST, 1:100), and RPS15A (TA369533S, ORIGENE, 1:50) followed by 30 min of blocking with goat serum (ZLI‐9021, ZSBIO) at 37°C. The slides were then dried overnight at 4°C. The next step was to cure the samples for 45 min at 37°C with biotinylated secondary antibodies. A 30 min incubation with HRP‐conjugated streptavidin followed by development with DAB (ZLI‐9018, ZSBIO) was administered to the slides. The last step was to mount, dehydrate, and counterstain the sections with hematoxylin. In order to analyze the protein expression, a microscope (Eclipse 8i, Nikon) was used.

### 2.14 Cell proliferation assay

We used CCK‐8 and colony formation assays to measure cell proliferation. The transfected cells were loaded into 96‐well plates with a density of 2–3 × 10^3^ cells/well in order to conduct the CCK‐8 test. Incubation was carried out for 3 h at 37°C after adding the CCK‐8 solution (K1018, APExBIO) at 0, 24, 48, 72, and 96 h. Microplate spectrophotometer (Bio‐Rad) absorbance measurements were taken at 450 and 630 nm. The six‐well plates were seeded with 2–3 × 10^3^ cells for the colony formation experiment, and the media was changed every 4 days. Following an 8–12‐day incubation period, the cells were fixed with 4% paraformaldehyde, stained with 0.1% crystal violet, rinsed with PBS, and subsequently counted using a light microscope.

### 2.15 Cell cycle assay

From the cell cycle kit (C1052, Beyotime), cells were collected and washed twice with PBS. The collected cells were fixed at 70% alcohol at 4°C for 24 h, propidium iodide staining solution was added, and incubated in a warm bath at 37°C away from light for 30 min. Finally, it is tested on the flow cytometer (BD Acurri C6). Modfit 5 software was used to analyze the results.

### 2.16 Transwell assay

To evaluate migration and invasion, 8 × 10^4^ cells were added to 200 µL of serum‐free media in transwell chambers (8 µm, Corning Falcon). For invasion experiments, matrigel coating was utilized and 600 mL of medium containing ten percent FBS was added to the lower chamber. After 24 h, three randomly selected fields were used to count the cells on the bottom side of the membrane after staining them with crystal violet.

### 2.17 Wound‐healing assay

The cells were grown in six‐well plates until they reached confluence after being seeded with full media. Using a regular 200 µL pipette tip, a straight incision around 300–500 µm broad was made. The non‐adherent cells were removed from the wounded monolayers by washing them twice with 1xPBS. At two prearranged intervals (0 and 12 h), the wound closure was photographed and recorded. The wound healing was measured as described: 12 h migration % = (0 h width – 12 h width of the wound)/(0 h width of the wound).

### 2.18 Xenograft mice experiment

Chongqing Medical University's Institutional Animal Care and Use Committee gave its approval to all animal experiments. The tumorigenesis assay involved injecting 5 × 10^6^ OVCAR3 cells subcutaneously into the right armpit region of female BALB/c nude mice that were 6 weeks old. We measured the tumour's width and length every three to four days. The mice were sacrificed 28 days after injection, and the weight of the tumours was noted. The formula *V* = .5×*A*×*B* ^2^ was used to get the tumour volume, with A and B standing for the longitudinal and latitudinal diameters, respectively. Furthermore, in order to facilitate further experiments, tumour tissues were embedded in 4% formalin. Nude mice were injected intraperitoneally with 5 × 10^5^ OVCAR3 cells transduced with shRNAs or empty vectors, which were suspended in 300 µL of PBS for the metastatic tests. The mice were euthanized after 3 weeks, and the number of metastatic nodules was determined.

### 2.19 RNA immunoprecipitation

A lysis solution containing RNA immunoprecipitation (RIP), DTT, PIC, and RNase inhibitor was used to lyse about 2 × 10^7^ cells. The buffer contained 20 mM HEPES, 150 mM NaCl, 10 mM KCl, 5 mM EDTA, 5 mM MgCl_2_, 0.5% NP40, and 10% glycerol. Centrifuged after 30 min of ice incubation, 5% of the supernatant was set aside as the input sample. Overnight at 4°C, the lysate was incubated with 3 µg of specific or control IgG antibody. The mixture was incubated with rotation at 4°C for 4 h the following day after adding 50 µL of washed protein G beads (Invitrogen, 10004D). To provide quality control, 10% of the immunoprecipitated material was set aside for western blot after five 5 min washes. TRIzol reagent (Invitrogen) was used to extract the RNA that co‐precipitated with the beads and input RNA. Then, ethanol was used to precipitate the mixture while glycogen was present. Following this, the standard operating protocol for RT‐qPCR analysis was followed.

### 2.20 m^6^A RNA immunoprecipitation

Magna MeRIP m^6^A kit (Millipore) was used to carry out the MeRIP experiment. First, cells were lysed in RIP buffer. Then, either anti‐m^6^A antibody (Abcam) or control IgG antibodies were used for immunoprecipitation overnight at 4°C. Finally, the RNA was purified. The RT‐qPCR analysis was performed on the immunoprecipitated RNA.

### 2.21 RNA stability assay

Actinomycin D (AbMole) was added to transfected OVCAR3 or SKOV3 cells in a 12‐well plate at a concentration of 5 µg/mL for 0, 2, 4, and 6 h intervals. After extraction, total RNA was reverse‐transcribed and evaluated using RT‐qPCR. As a means of measuring RNA abundance, the average Ct value at each time point was set to 0 h. GraphPad Prism 9.5 was used for nonlinear regression curve fitting, namely one phase decay, in order to establish the RNA half‐life.

### 2.22 RNA fluorescence in situ hybridization

OVCAR3 or SKOV3 cells were subjected to RNA fluorescence in situ hybridization (FISH) with probes specific to human RPS15AP12 (RiboBio). RiboBio developed Cy3‐labeled RPS15AP12 probes that were custom‐designed. As internal controls for nuclear and cytoplasmic localization, respectively, FISH probes for human U6 snRNA and 18S rRNA were utilized.

### 2.23 Luciferase reporter assay

Co‐transfection of pmirGLO, pmirGLO‐WT for RPS15A, and pmirGLO‐MUT for RPS15A constructs with miR‐96‐3p mimics or an RPS15AP12 overexpression plasmid was performed using jetPRIME reagent (Polyplus) into OVCAR3 or HEK293T cell lines. After 48 h of transfection, the relative luciferase activity was determined using a double‐luciferase reporter assay kit (TransGen) and adjusted to Renilla luciferase activity. The results were shown as a fold change compared with the control groups that were matched, and each experiment was carried out three times.

### 2.24 Statistic methods

#### 2.24.1 Survival analysis with the Cox proportional hazards model

Using the Cox proportional hazards regression (Cox) model, the RPKMs of RNA in the TCGA OC cohort were examined for OS and progression‐free survival (PFS). This allowed us to examine the predictive role of each pseudogene RNA. In terms of correlation with PFS/OS, a *p*‐value less than or equal to.05 was deemed significant. Risk factors were genes with an HR greater than 1, whereas protective factors were genes with an HR less than 1.

#### 2.24.2 Statistical comparison

Data were expressed as mean ± SEM or SD. Two‐tailed Student's *t*‐tests were conducted to evaluate the statistical significance of the differences observed between the two groups. Wilcoxon tests were performed to evaluate the significance of differences among various groups. The Spearman correlation was employed to examine the relationships between variables. *p* < .05 was considered statistically significant. All statistical analyses were performed using GraphPad Prism 8.0 or R software (version 3.6.1). *p < .05, **p < .01, ***p < .001; ns, not significant.

## 3 RESULTS

### 3.1 Integrative multi‐omics analysis reveals dysregulation of pseudogenes in OC

**FIGURE 1:**
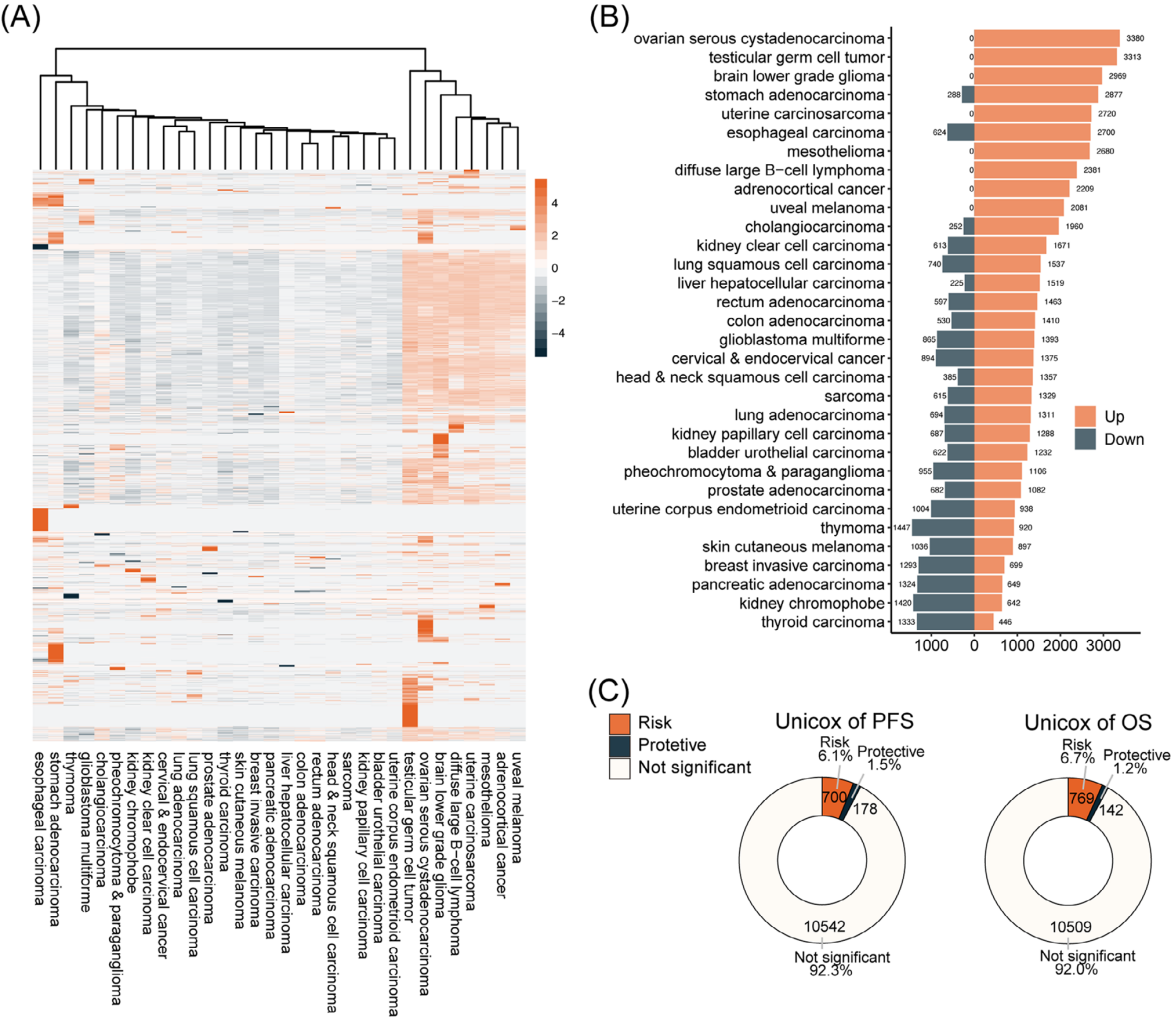
Pervasive dysregulation of pseudogenes in ovarian cancer. (A) Heatmap of DEPGs fold changes of TCGA vs. GTEx RNA‐seq datasets in Pan‐cancer (left). (B) Bar plot of DEPG numbers of TCGA vs. GTEx RNA‐seq datasets (right). Orange, upregulated in tumour specimens; dark blue, upregulated in normal control specimens. (C) Univariable Cox regression analysis results of OS and PFS. Risk, according to the expression of pseudogenes, pseudogenes that have HR > 1 and *p*‐value < .05 are considered risk factors; protective, pseudogenes that have HR < 1 and *p*‐value < .05 are considered protective factors; not significant, univariable Cox regression analysis implicated no statistical significance.

Initially, a landscape analysis was conducted to evaluate the differential expression of pseudogenes in Pan‐cancer compared with the corresponding normal controls. Approximately 1000–2000 pseudogenes were dysregulated in Pan‐cancer, with a significant number upregulated in OC (Figure 1A,B). It was speculated that elevated expression of pseudogenes might trigger functional activation, and subsequently, univariate Cox analysis was performed to validate this prediction. According to the TCGA‐OV cohort of RNA‐seq, 911 and 878 pseudogenes were identified with prognostic values for OS and PFS, respectively, with higher expression correlating to poorer survival in most cases (Figure 1C).

Next, we investigated the genetic alterations that triggered the dysregulation of pseudogenes. Among 1246 differentially expressed pseudogenes (DEPGs) identified in HGSOC (Figure S1A), genetic changes were observed in only 73 pseudogenes, affecting less than 6% of patients. Of these, only two pseudogenes exhibited mutation frequencies of less than 1%, and four of 72 patients with copy number variation (CNV) had frequencies higher than 5% (Figure S1B). Among the pseudogenes exhibiting the highest frequencies of CNV, TSPY26P, and RPL13P5 showed a positive correlation between CNV and RNA expression (Figure S1C). Nevertheless, no significant correlation was found between their expression and patient survival outcomes (Figure S1D). Landscape statistics of 12 146 pseudogenes for genetic alteration were also conducted, showing that nearly all pseudogenes underwent negligible frequencies of genetic mutation (Figure S1E) and only 6 pseudogenes had frequencies of CNV greater than 10% (Figure S1F). Among those pseudogenes with high frequencies of genetic alteration, only the expression of eight pseudogenes were influenced (Figure S1G). A correlation was noted between the expression of a few pseudogenes and both gene copy number and OS (Figure S1H,I). These results demonstrate the dysregulation of pseudogenes in HGSOC, which may not be primarily due to genomic causes.

### 3.2 Epitranscriptome‐wide profiling of m^6^A modification in pseudogene‐derived RNAs

**FIGURE 2:**
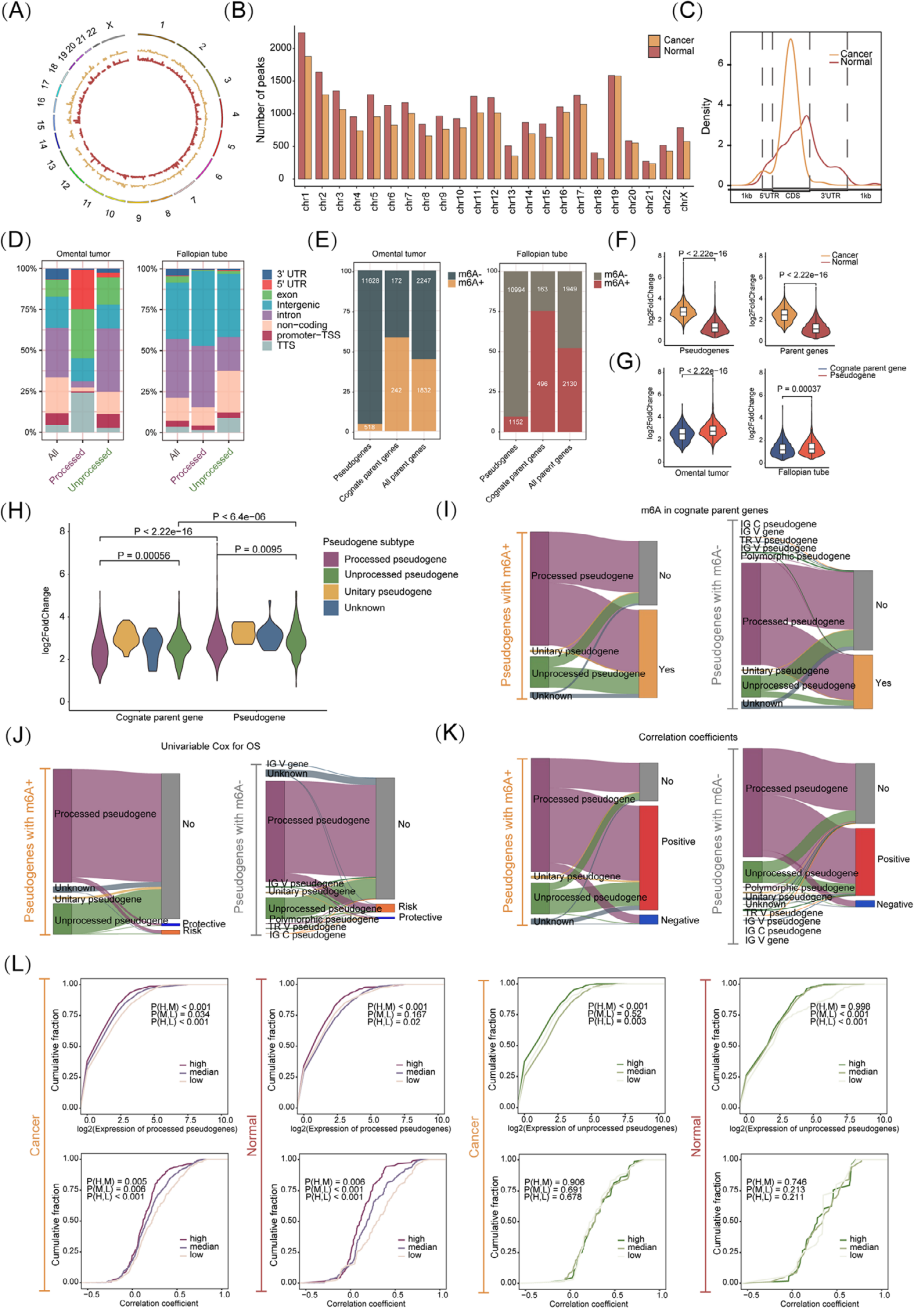
Epitranscriptome‐wide profiling of m^6^A modification in pseudogene‐derived RNAs. (A) Circos plot showing the chromosome distribution of m^6^A peaks on pseudogene RNAs. (B) Numbers of m^6^A peaks on pseudogene RNA on 23 chromosomes. (C) Guitar plot displaying gene region distribution of pseudogene m^6^A peaks in cancer and normal group. (D) Annotation of pseudogene m^6^A peaks in gene region by HOMER in cancer and normal group as well as the subgroup analysis in processed and unprocessed pseudogenes. (E) Number of pseudogenes that have m6A peaks among 12 146 pseudogenes, cognate parent genes that have m^6^A modification among those corresponding pseudogenes with m^6^A modification as well as all those with m^6^A modification among 4078 paired parent genes, in omental tumour and fallopian tube tissues separately. (F) Comparisons of m^6^A peak log2(fold change) in omental tumour and fallopian tube tissues between pseudogenes and cognate parent genes. (G) Comparisons of m^6^A peak log2(fold change) between pseudogenes and cognate parent genes in omental tumour and fallopian tube tissues. (H) Subgroup analysis of comparisons m^6^A peak log2(fold change) of pseudogene subtype between pseudogenes and cognate parent genes. (I) Sankey plot showing the correspondence between pseudogenes with (left) and without m^6^A modification (right) and m^6^A modification on their cognate parent genes. (J) Sankey plot showing the correspondence between pseudogenes with (left) and without m^6^A modification (right) and the relevance of their expression with OS. (K) Sankey plot showing the correspondence between pseudogenes with (left) and without m^6^A modification (right) and the correlation of their expression with their cognate parent genes. Positive, Spearman correlation coefficient <0 and *p*‐value < .05; Negative, Spearman correlation coefficient <0 and *p*‐value < .05; No, Spearman correlation analysis implicated no statistical significance. (L) CDF plot of unprocessed and processed pseudogene expression in high, median and low m^6^A level groups. Wilcox analysis was applied in comparisons between multiple groups.

To discover the mechanism underlying the pseudogenes dysregulation in OC, we focused on epigenetic modulation. Since 72% of pseudogenes are processed through retro‐transposition of mRNA transcripts, lacking introns, they are not subject to NMD RNA surveillance.^11^ These NMD‐resistant processed pseudogenes acquire de novo m^6^A sites under positive natural selection, establishing m^6^A‐mediated degradation as a crucial RNA surveillance mechanism.^11^ We examined m^6^A modification in pseudogenes across the genome using MeRIP data from HGSOC clinical specimens. m^6^A peaks are widely distributed across pseudogenes on all chromosomes, with a higher concentration on chromosome 1 (Figure 2A,B). Additionally, more m^6^A peaks were located in the coding sequence region (Figure 2C). HOMER analysis indicated that processed pseudogenes predominantly exhibited more m^6^A peaks in the exon region of omental tumour tissues, while more m^6^A peaks were found in the intron and intragenic regions in fallopian tube epithelium tissues used as normal controls (Figure 2D). We also analyzed the prevalence of m^6^A modification in pseudogenes in tumour and normal tissues, finding that fewer pseudogenes were m^6^A‐modified in tumour tissues (Figure 2E). Similarly, fewer corresponding cognate parent genes of those m^6^A‐modified pseudogenes showed modifications in tumour tissues and all 4078 paired parent genes from 12 146 pseudogenes (Figure 2E). Moreover, a higher abundance of m^6^A peaks was observed in fewer pseudogenes in tumours compared with controls (Figure 2F). Despite the generally low expression of ncRNAs, a higher abundance of m^6^A modification was observed in pseudogenes compared with their cognate parent genes (Figure 2G). These results support a redistribution and potential function of m^6^A modification in pseudogene‐derived RNAs.

According to their origin, pseudogenes are classified into four types: processed, unprocessed, unitary, and polymorphic.^2^ Unitary pseudogenes exhibited the highest abundance of m^6^A, while unprocessed pseudogenes had a higher abundance than processed pseudogenes (Figure 2H). We then used a Sankey Plot to visualize the landscape of m^6^A‐positive and m^6^A‐negative pseudogenes, and their correspondence to function and potential interplay with their cognate parent genes. Pseudogenes with m^6^A modifications had a higher proportion of m^6^A modifications in parent genes in OC, suggesting that more pseudogenes might retain those m^6^A sites from their cognate parent genes during evolution (57.9% vs. 40.3%, *p* < .001; Figure 2I). Through univariate Cox regression analysis, a higher expression of the majority of pseudogenes was correlated with poorer OS, regardless of pseudogene type and m^6^A modification (Figure 2J). Positive correlations were predominantly observed in most both m^6^A‐positive and m^6^A‐negative pseudogenes of expression with their paralogs, from which we speculated that ceRNA regulation could serve as a dominant posttranscriptional regulatory mechanism between pseudogenes and their paralogs (Figure 2K). We analyzed pseudogene expression across different m^6^A levels in two major subtypes, finding similar m^6^A‐mediated down‐regulation; both processed and unprocessed pseudogenes had m^6^A negatively correlated with expression as shown by the cumulative distribution function (CDF) plot (Figure 2L).

To evaluate the influence of m^6^A modification on the relevance between pseudogenes and their paralogs, we analyzed the correlation coefficients of gene expression between pseudogenes and cognate parent genes at varied levels of m^6^A modification in pseudogenes. Processed pseudogenes exhibiting elevated m^6^A levels demonstrated markedly reduced correlation coefficients, a trend that was not evident in unprocessed pseudogenes (Figure 2L). As controls, lncRNAs and mRNAs both showed m^6^A negatively correlated with expression, and higher m^6^A levels correlated with lower correlation coefficients (Figure S2). Overall, pseudogene subtypes could trigger varied m^6^A effects.

### 3.3 Identification of the critical Diff‐m^6^A pseudogene RNAs in OC

**FIGURE 3:**
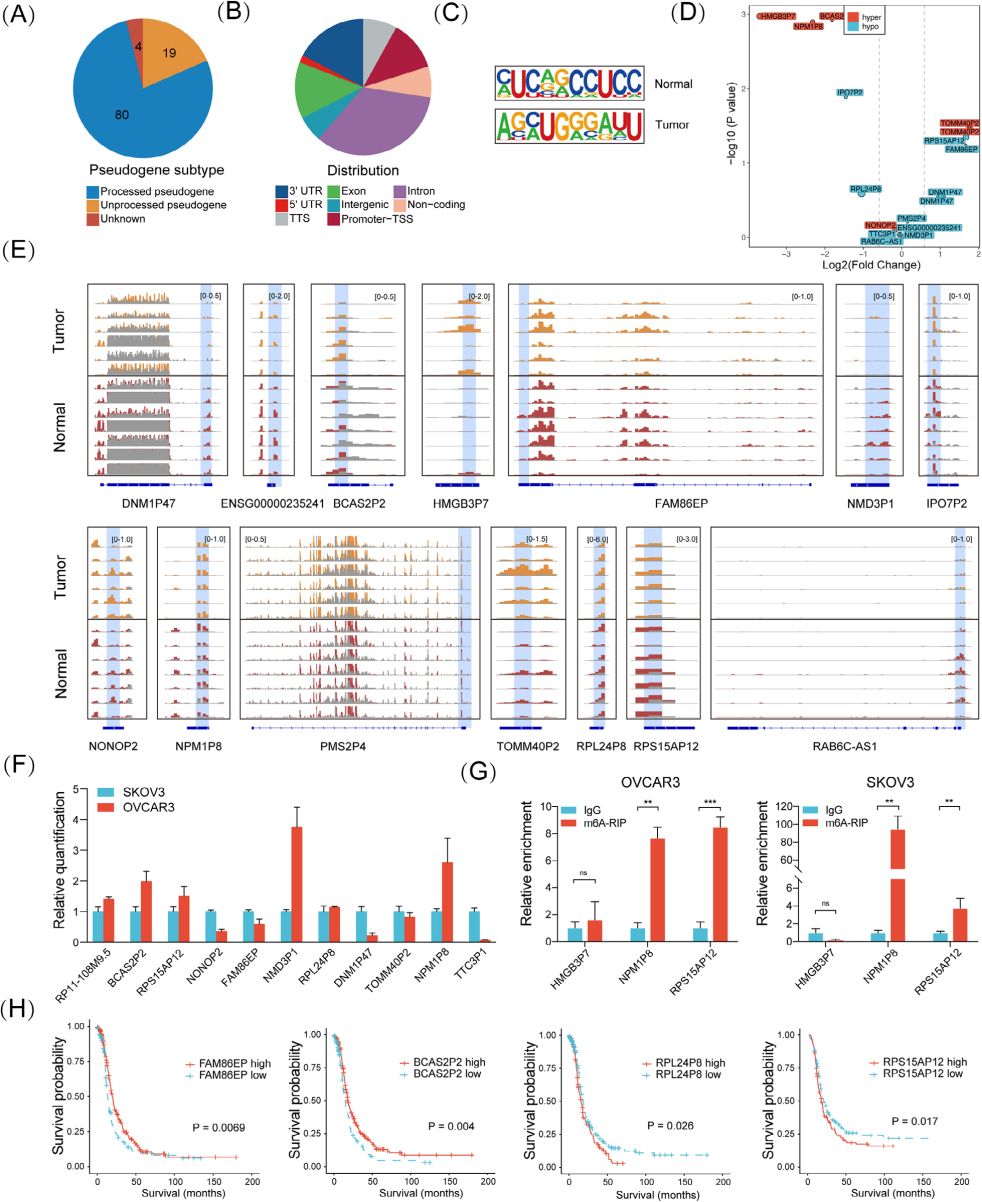
Identification of critical differential‐m^6^A pseudogenes in ovarian cancer. (A) Numbers of pseudogenes that have a differential‐m^6^A modification in each subtype. (B) Annotation of pseudogenes with differential‐m^6^A modification in the gene region. (C) Motifs identified by MEME in pseudogene differential‐m^6^A sites in normal and tumour specimens. (D) Volcano plot showing log2(fold change) and *p*‐value of corresponding differential expression of differential‐m^6^A pseudogenes. Hyper, hypermethylated of m^6^A in tumour specimens; hypo, hypo‐methylated of m^6^A in tumour specimens. (E) The differential m^6^A sites of representative pseudogenes in tumour and normal samples. (F) RT‐qPCR of differential‐m^6^A pseudogenes in OVCAR3 and SKOV3 cell lines. (G) MeRIP‐PCR of HMGB3P7, NPM1P8, and RPS15AP12. (H) Kaplan–Meier survival analysis of FAM86EP, BCASP2, RPL24P8 and RPS15AP12 expression according to the TCGA‐OV dataset.

We aimed to identify critical m^6^A‐regulated pseudogenes with predictive and therapeutic potential. A total of 103 pseudogenes with differential m^6^A (Diff‐m^6^A) levels in HGSOC compared with fallopian epithelium tissues were identified, with processed pseudogenes comprising 77.7% (Figure 3A). Most Diff‐m^6^A sites were located in the intron, exon, and 3′UTR regions (Figure 3B). The typical RRACH motif (R = G or A; H = A, C, or U) was identified using MEME motif analysis (Figure 3C). Integrative m^6^A and gene expression analysis revealed that 15 pseudogenes exhibited significant differential m^6^A and stabilized RNA levels, with seven showing concurrent differential expression (Figure 3D). An IGV plot illustrated the differential m^6^A modification levels of representative pseudogenes (Figure 3E). RT‐qPCR was conducted to confirm their expression in OC cells (Figure 3F). MeRIP‐PCR confirmed the m^6^A modification of NPM1P8 and RPS15AP12 in OC cells (Figure 3G). Genomic profiling supported that RNA expression changes in these pseudogenes were not primarily due to genetic alterations, as very low frequencies of mutation and CNV ranging from 2% to 7% were present among only four pseudogenes (Figure S3A). Kaplan–Meier survival analyses showed that FAM86EP, BCASP2, RPL24P8, and RPS15AP12 had significant predictive value for survival (Figure 3H). Notably, RPS15AP12 was the highest ranked among up‐regulated Diff‐m^6^A pseudogenes in TCGA‐OV versus GTEx (Figure S3B). Thus, a set of m^6^A‐regulated pseudogenes potentially implicated in OC was identified.

### 3.4 Pseudogene RPS15AP12‐lncRNA promotes OC progression

**FIGURE 4:**
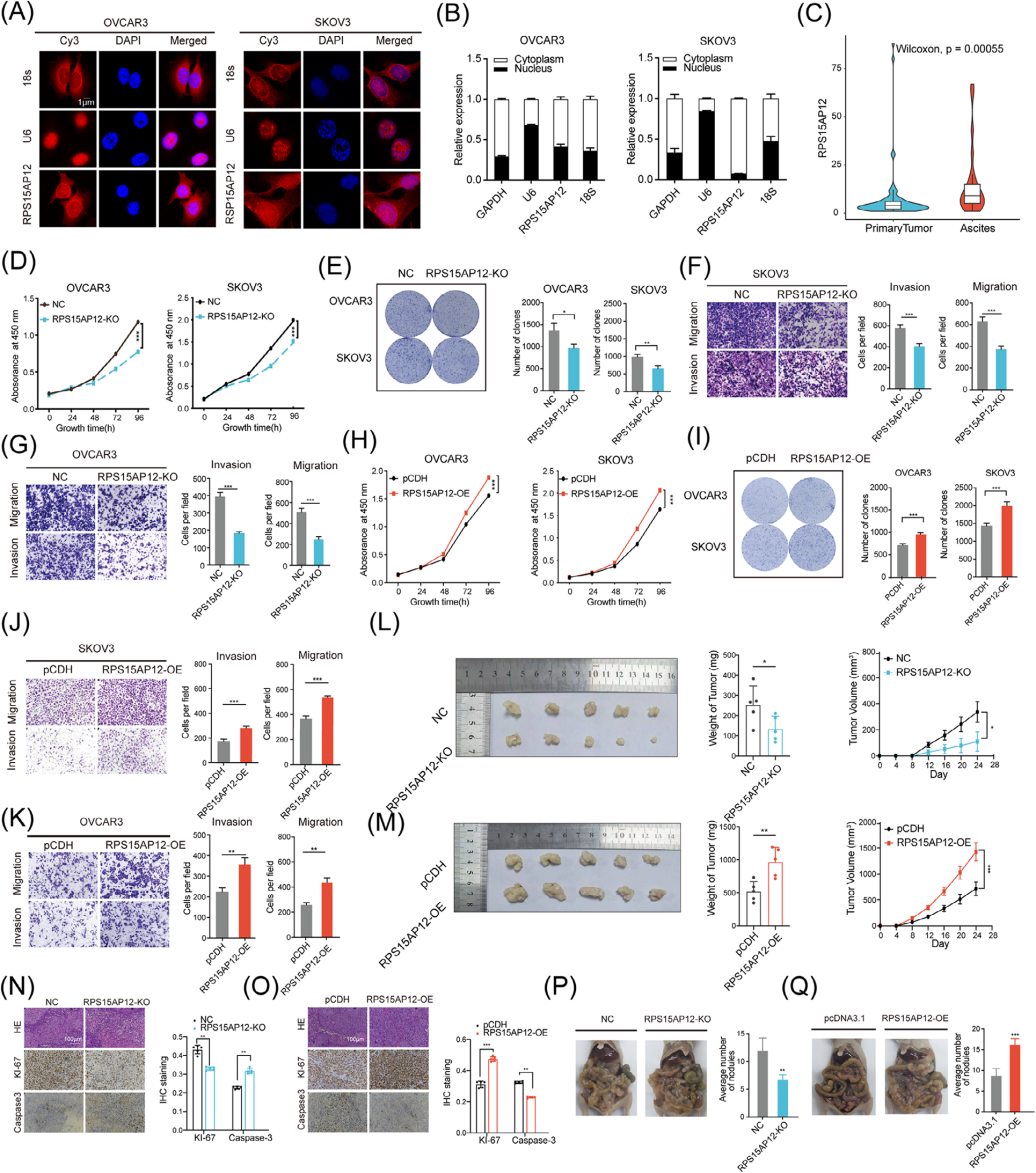
Pseudogene RPS15AP12‐lncRNA promotes ovarian cancer progression. (A) RNA FISH assay of RPS15AP12‐lncRNA in OVCAR3 and SKOV3 cells. (B) RPS15AP12 RNA in nuclear and cytoplasmic fractions of OVCAR3 and SKOV3 cells. (C) Boxplot of RPS15AP12 expression in serous ovarian cancer tumour and ascites samples in GSE102073 dataset. (D) CCK‐8 assays of OVCAR3 and SKOV3 cells upon RPS15AP12 knockout. (E) Colony formation assays were performed in OVCAR3 and SKOV3 cells upon RPS15AP12 knockout. (F, G) Transwell assays detecting migration and invasion of OVCAR3 and SKOV3 cells with RPS15AP12 knockout. (H) CCK‐8 assays of OVCAR3 and SKOV3 cells upon RPS15AP12 overexpression. (I) Colony formation assays were performed in OVCAR3 and SKOV3 cells upon RPS15AP12 overexpression. (J, K) Transwell assays detecting migration and invasion of OVCAR3 and SKOV3 cells with RPS15AP12 overexpression. (L, M) Effects of RPS15AP12 KO and RPS15AP12 OE on tumour weight and volume in the subcutaneous xenograft nude mouse model. (N, O) Representative IHC staining images and quantitative analysis of Ki‐67 and Caspase‐3 in xenograft tumours from RPS15AP12‐KO and RPS15AP12‐OE cells treated nude mice. Scale bar, 100 µm. Statistical analyses showed the IHC staining of Ki‐67 and Caspase‐3 from xenograft tumours. (P, Q) The metastasis of OVCAR3 and SKOV3 cells with or without RPS15AP12 KO and RPS15AP12 OE to the peritoneal cavity of mice was assessed. One‐way ANOVA, *p < .05, **p < .01, ***p < .001; NS, not significant.

To address the role of m^6^A‐regulated pseudogenes in OC further, we focused on the up‐regulated Diff‐m^6^A pseudogene RPS15AP12, a processed pseudogene derived from the mRNA reverse transcription of RPS15A (Figure S4A). To determine whether RPS15AP12 is translated into a protein, an Open Reading Frame (ORF) prediction was performed. Intriguingly, a potential ORF of 118 amino acids was identified in RPS15AP12 (Figure S4B). However, FLAG‐tagging assays of the putative protein revealed that no corresponding proteins were detected in cells transfected with the RPS15AP12 ORF‐FLAG fusion vector, suggesting RPS15AP12 might function as a long noncoding RNA (Figure S4C). Cellular fractionation and RNA‐FISH assays revealed that RPS15AP12‐lncRNA predominantly resides in the cytoplasm (Figure 4A,B).

In addition to its high expression in OC, RPS15AP12 was also overexpressed in the ascites of OC patients (Figure 4C), indicating its potential relevance to invasiveness. RT‐PCR also confirmed that RPS15AP12 had higher expression in the OC samples than in the FTE samples (Figure S4D). To investigate the biological functions of RPS15AP12‐lncRNA in OC, we knocked out the RPS15AP12 pseudogene in OC cells using the CRISPR‐Cas9 genome‐editing tool (Figure S4E,F). The growth, migration, invasion, and colony formation of OC cells were all dramatically reduced when RPS15AP12 was depleted (Figures 4D–G; Figure S4G) and the cell cycle of OC cells was changed (Figure S4I,J). Likewise, these carcinogenic tendencies were enhanced by the forced expression of RPS15AP12 (Figures 4H–K; Figure S4H). Using a subcutaneous xenograft model and a peritoneal metastasis model, we observed that depletion of RPS15AP12 substantially reduced subcutaneous xenograft formation both in volume and weight by OC cells, as well as the metastasis of xenograft tumours (Figure 4L,P). Forced expression of RPS15AP12 enhanced subcutaneous xenograft formation and metastasis (Figure 4 M,Q). Furthermore, immunohistochemistry (IHC) staining showed that KO of RPS15AP12 decreased the protein level of Ki‐67 but increased the protein level of Caspase‐3 in subcutaneous xenografts, whereas overexpression of RPS15AP12 had the opposite effects (Figure 4N,O). These results demonstrate that RPS15AP12‐lncRNA facilitates OC progression.

### 3.5 FTO‐mediated demethylation of m^6^A contributes to upregulation of RPS15AP12‐lncRNA in OC cells

**FIGURE 5:**
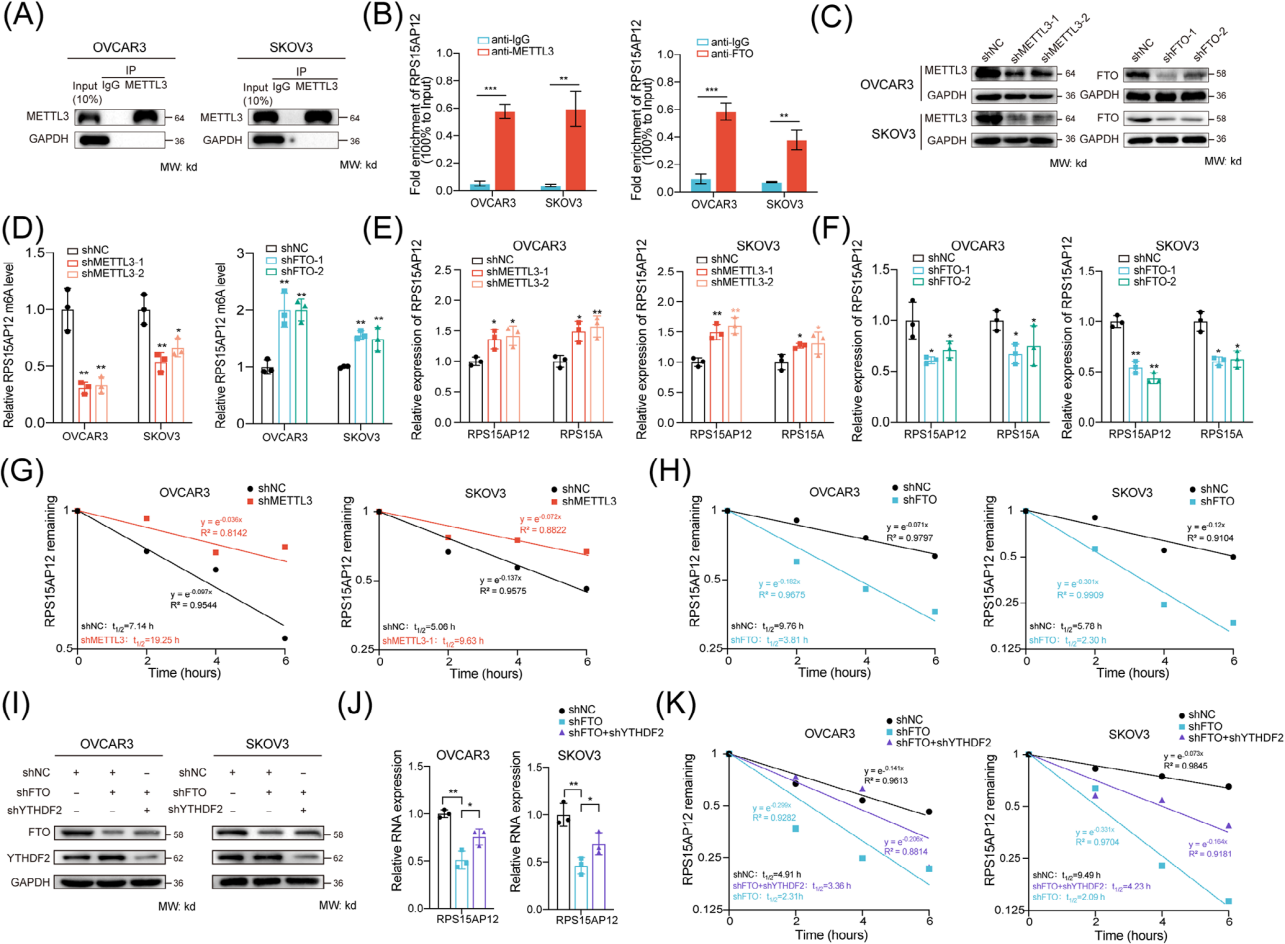
m^6^A‐mediated down‐regulation of RPS15AP12‐lncRNA. (A, B) RIP‐PCR assays detecting interaction of FTO and METTL3 with RPS15AP12‐lncRNA in OVCAR3 and SKOV3 cell lines. IgG was used as an internal control. (C) Western blot detecting protein levels of METTL3 and FTO in OVCAR3 and SKOV3 cell lines upon METTL3 and FTO knockdown. GAPDH was used as the negative control in western blot assays. (D) MeRIP‐PCR detecting m^6^A level of RPS15AP12 upon METTL3 and FTO knockdown. (E, F) RT‐qPCR assays detecting the expression of RPS15AP12‐lncRNA upon METTL3 and FTO knockdown in OVCAR3 and SKOV3 cell lines. (G, H) RNA half‐life assays detecting the stability of RPS15AP12‐lncRNA upon FTO and METTL3 knockdown. (I) Western blot detecting protein levels of FTO and YTHDF2 in OVCAR3 and SKOV3 cell lines upon FTO knockdown, YTHDF2 knockdown and FTO+YTHDF2 knockdown. (J) RT‐qPCR assays detecting the expression of RPS15AP12‐lncRNA upon FTO knockdown, YTHDF2 knockdown and FTO+YTHDF2 knockdown in OVCAR3 and SKOV3 cell lines. (K) RNA half‐life assays detecting the stability of RPS15AP12‐lncRNA upon FTO knockdown, YTHDF2 knockdown and FTO+YTHDF2 knockdown. One‐way ANOVA, *p < .05, **p < .01, ***p < .001; NS, not significant.

Integrative analysis indicated that m^6^A hypermethylation tended to reduce transcript levels in HGSOC compared with normal tissues, though not statistically significant (Figure S5A). Since RPS15AP12 was observed to be hypomethylated and upregulated in OC (Figure 3D), we investigated whether m^6^A modification regulated RPS15AP12. The Spearman correlation analysis of expression showed that RPS15AP12 was positively correlated with FTO and negatively correlated with METTL3 and YTHDF2 (Figure S5B), but no significant correlation was found between RPS15AP12 with ALKBH5 and METTL14 (Figure S5B). RIP assays using antibodies specific to METTL3 and FTO demonstrated that both could bind to RPS15AP12‐lncRNA (Figure 5A,B). meRIP‐PCR results showed that METTL3 knockdown decreased m^6^A modification on RPS15AP12‐lncRNA, while FTO knockdown increased it (Figure 5C ,D). Upon METTL3 or FTO depletion in OC cells, RPS15AP12‐lncRNA expression increased and decreased, respectively (Figure 5E,F). RNA decay assays indicated that METTL3 knockdown shortened the half‐life of RPS15AP12‐lncRNA, whereas FTO knockdown enhanced its stability (Figure 5G,H). YTHDF2, known as an m^6^A reader involved in RNA degradation, was found to increase both expression and stability of RPS15AP12 when FTO was knocked down, suggesting YTHDF2's involvement in m^6^A‐mediated degradation of RPS15AP12‐lncRNA (Figure 5I–K; Figure S5C–E).

### 3.6 RPS15AP12‐lncRNA acts as a miRNA sponge to positively regulate RPS15A expression

**FIGURE 6:**
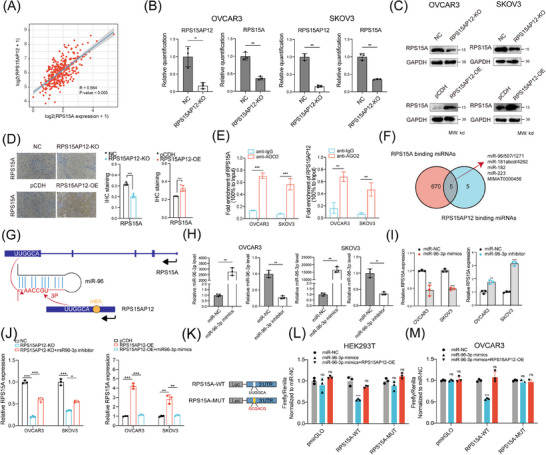
RPS15AP12‐lncRNA competitively combines miR‐96‐3p to positively regulate RPS15A expression. (A) Spearman's correlation analysis between RNA levels of RPS15AP12 and RPS15A according to the TCGA ovarian cancer cohort. (B) RT‐qPCR assays detecting the expression of RPS15A upon RPS15AP12 KO in OVCAR3 and SKOV3 cell lines. (C) Western blot detecting the protein level of RPS15A upon RPS15AP12 KO in OVCAR3 and SKOV3 cell lines. (D) Representative IHC staining images and quantitative analysis of RPS15A in xenograft tumours from RPS15AP12 knockout and control cells treated nude mice. Scale bar, 100 µm. Statistical analyses showed the IHC staining of RPS15A from xenograft tumours. (E) AGO2 RIP‐PCR detecting the binding of RPS15A and RPS15AP12 with miRNAs. (F) Venn plot showing co‐binding miRNAs shared by RPA15A and RPS15AP12. (G) Schematic diagram of the binding site and sequence in miR‐96, RPS15AP12 and RPS15A. RT‐qPCR detecting miR‐96‐3p level upon miR‐96‐3p mimics and miR‐96‐3p inhibitor in OVCAR3 and SKOV3 cell lines. (H) RT‐qPCR detecting miR‐96‐3p expression upon miR‐96‐3p mimics and miR‐96‐3p inhibitor in OVCAR3 and SKOV3 cell lines. (I) RT‐qPCR detecting RPS15A mRNA level upon miR‐96‐3p mimics and miR‐96‐3p inhibitor in OVCAR3 and SKOV3 cell lines. (J) RT‐qPCR detecting RPS15A mRNA level was performed with control, RPS15AP12‐KO, and RPS15AP12‐KO+miR‐96‐3p inhibitor in OVCAR3 and SKOV3 cell lines. RT‐qPCR detecting RPS15A mRNA level was performed with control, RPS15AP12‐OE, and RPS15AP12‐OE+miR‐96‐3p mimics in OVCAR3 and SKOV3 cell lines. (K) The schematic diagram of wild‐type and mutant 3′UTR of RPS15A for luciferase assays. (L, M) Luciferase assays of RPS15A‐WT and RPS15A‐MUT upon control, miR‐96‐3p mimics and miR‐96‐3p mimics+RPS15AP12‐OE in HEK293T and OVCAR3 cells. One‐way ANOVA, *p < .05, **p < .01, ***p < .001; NS, not significant.

RPS15AP12 was primarily localized in the cytoplasm (Figure 4A,B), implicating it may work as a miRNA sponge. The positive correlation between RPS15AP12 and RPS15A indicated potential ceRNA regulation (Figure 6A). RT‐qPCR and western blot analyses revealed that RPS15A expression decreased following RPS15AP12 KO (Figure 6B,C). IHC staining showed that RPS15AP12 KO significantly reduced RPS15A protein levels, whereas RPS15AP12 overexpression significantly increased them (Figure 6D). A RIP assay using an anti‐AGO2 antibody demonstrated that RPS15AP12, unlike control IgG, could bind with miRNAs (Figure 6E). Three bioinformatics databases (miRcode, TargetScan, and ENCORI) were utilized to predict miRNAs and their binding sites on RPS15A and RPS15AP12, revealing several potential co‐binding miRNAs (Figure 6F). Among these, miR‐96‐3p showed a negative correlation with RPS15A expression (Figure S6A), and its inhibition significantly increased RPS15A expression (Figure S6B). The focus then shifted to miR‐96‐3p's role in RPS15AP12‐lncRNA‐mediated upregulation of RPS15A. RPS15AP12 contains the sequence UUGGCA, shared with RPS15A, matching the seed region of miR‐96‐3p (Figure 6G). Experimentally, a miR‐96‐3p mimic reduced RPS15AP12 expression at the RNA level in OC cells, while a miR‐96‐3p inhibitor increased RPS15A expression (Figure 6H,I). In addition, the down‐regulation of RPS15A generated by RPS15AP12 KO could be undone by an inhibitor of miR‐96‐3p, while the upregulation of RPS15A caused by RPS15AP12 overexpression could be undone by miR‐96‐3p mimics (Figure 6J). To validate the binding of miR‐96‐3p to RPS15A, the 3′UTR region of RPS15A or a mutant version of it without the miR‐96‐3p binding site was inserted into a luciferase reporter vector. The results showed that luciferase activity was significantly reduced when co‐transfected with miR‐96‐3p, but it remained unaffected when the binding site was mutated (Figure 6K–M). All things considered, these results point to the competitive binding of RPS15AP12‐lncRNA to miR‐96‐3p as a mechanism by which RPS15A expression is enhanced.

To assess the impact of the RPS15AP12‐lncRNA‐miR‐96‐3p‐RPS15A axis on OC cell proliferation, RPS15AP12‐depleted OC cells were either overexpressed with RPS15A or treated with a miR‐96‐3p inhibitor. Depletion of RPS15AP12 significantly reduced colony formation in OC cells, but either re‐expression of RPS15A or inhibition of miR‐96‐3p lessened the suppression of cell proliferation caused by RPS15AP12 KO (Figure S6C). Comparably, the application of either a miR‐96‐3p inhibitor or the re‐expression of RPS15A resulted in a partial reversal of the inhibitory impact that RPS15AP12 KO had on the invasion and migration of OC cells (Figure S6D).

### 3.7 RPS15AP12‐lncRNA suppresses innate immune response in OC

**FIGURE 7:**
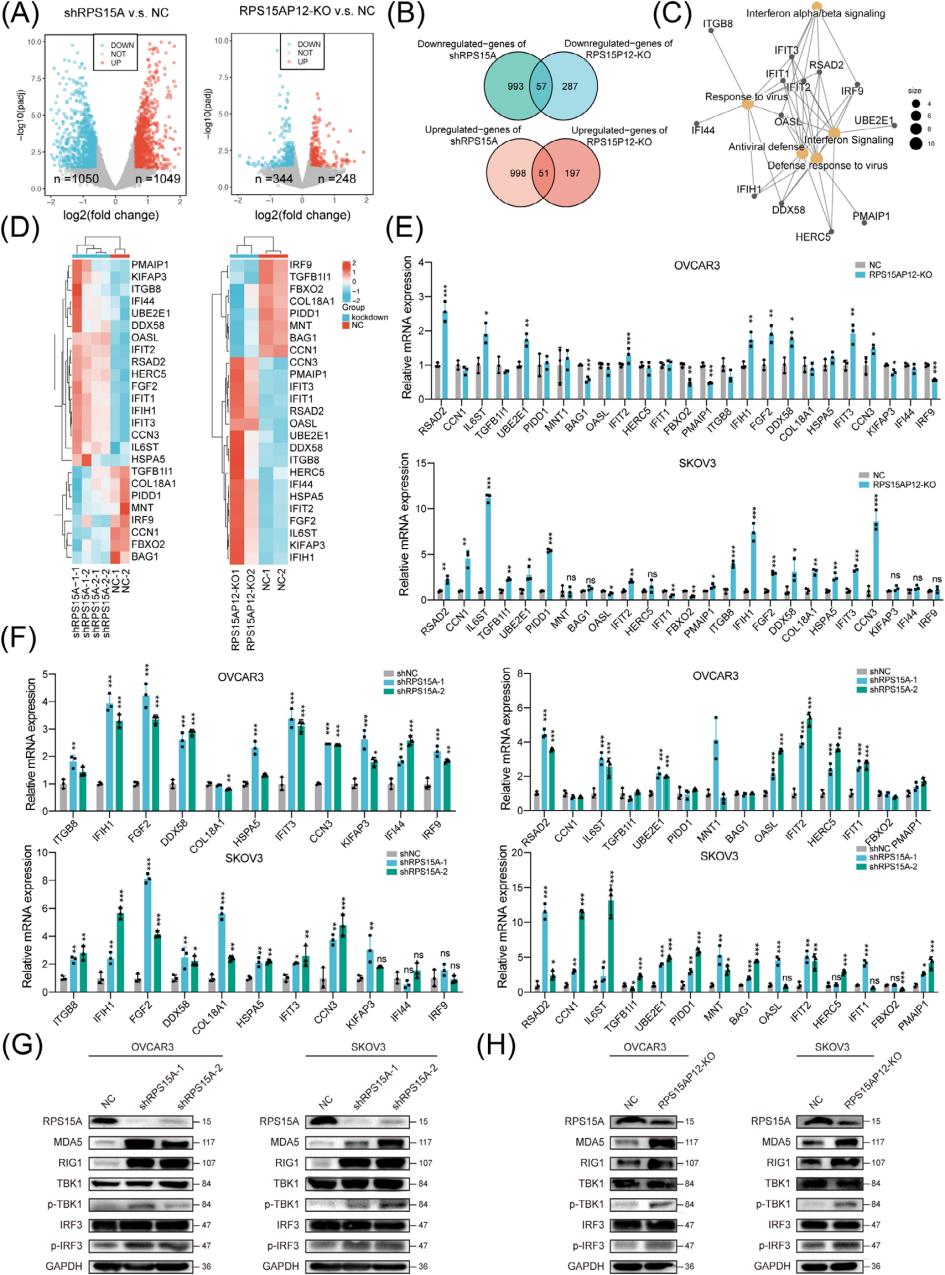
RPS15AP12 inhibits the anti‐tumour effect of innate immune‐related pathways in ovarian cancer. (A) Volcano plot showing log2(fold change) and *p*‐value of RNA‐seq upon RPS15A knockdown and RPS15AP12 knockout. (B) Overlapped DEGs of RNA‐seq upon RPS15A knockdown and RPS15AP12 knockout. (C) Enrichment network showing innate immune‐related pathways and corresponding hub genes by RPS15A knockdown and RPS15AP12 knockout. (D) Heatmap of 25 shared DEGs in innate immune‐related and proliferation‐related pathways in RNA‐seq upon RPS15A knockdown and RPS15AP12 knockout. (E) RT‐qPCR detecting RNA level of 25 shared DEGs in innate immune‐related and apoptosis‐related pathways in OVCAR3 and SKOV3 cell lines upon RPS15AP12 KO. (F) RT‐qPCR detecting RNA level of 25 shared DEGs in innate immune‐related pathways in OVCAR3 and SKOV3 cell lines upon RPS15A knockdown. (G) Western blot assays detecting protein expression using antibodies as indicated in OVCAR3 and SKOV3 cell lines upon RPS15A knockdown. (H) Western blot assays detecting protein expression using antibodies as indicated in OVCAR3 and SKOV3 cell lines upon RPS15AP12 depletion. One‐way ANOVA, *p < .05, **p < .01, ***p < .001; NS, not significant.

As expected, RPS15A was overexpressed in HGSOC compared with normal controls (Figure S7A), and RT‐qPCR assays also confirmed its high expression as well as its strong positive correlation with RPS15AP12 expression in OC (Figure S7B,C). Besides, higher expression of RPS15A was correlated with poorer survival of OC patients (Figure S7D,E). Therefore, RPS15A could be regulated by RPS15AP12 to exhibit an oncogenic role either. Analogous to the KO of RPS15AP12, the knockdown of RPS15A markedly diminished the proliferation, migration, and invasion of OC cells and changed cell cycles (Figure S7F–L). Overexpression of RPS15A promoted the migration of OC cells (Figure S7M,N). Knockdown of RPS15A could also impair the ability of subcutaneous tumour formation and intraperitoneal metastasis of OC cells in xenografts (Figure S7O,Q). On the contrary, overexpression of RPS15A enhanced the ability of subcutaneous tumour formation and intraperitoneal metastasis of OC cells in xenografts (Figure S7P,R). To investigate the downstream targets of RPS15AP12 and RPS15A in OC cells, RNA sequencing was conducted after the knockdown of RPS15A or the KO of RPS15AP12. Knockdown of RPS15A caused the upregulation of 1049 genes and the downregulation of 1050 genes. In contrast, the KO of RPS15AP12 resulted in 248 upregulated genes and 344 downregulated genes (Figure 7A). The overlapping analysis identified 51 upregulated genes and 57 downregulated genes common to both RPS15AP12 KO and RPS15A knockdown (Figure 7B). Function enrichment analysis revealed that the differentially expressed genes (DEGs) exhibited significant enrichment in pathways associated with cancer and innate immunity (Figure S7S). Further enrichment network analysis revealed hub genes involved in innate immune‐related pathways including IFN signalling, response to viruses, and IFN‐α/β signalling, as well as links between them (Figure 7C). A heatmap displayed the expression patterns of 25 shared DEGs related to apoptosis, proliferation, and innate immunity (Figure 7D). RT‐qPCR confirmed significant upregulation of RSAD2, IL6ST, UBE2E1, IFIT2, IFIH1, FGF2, DDX58, IFIT3, and CCN3 following either RPS15AP12 KO or RPS15A knockdown (Figure 7E,F). DDX58 (RIG‐I) and IFIH1 (MDA5) are RNA sensors upstream of MAVS, whose activation triggers downstream IFN signalling pathways. The examination of RIG‐I and MDA5 expression, along with the phosphorylation levels of TBK1 and IRF3, revealed an increase in response to either RPS15AP12 KO or RPS15A knockdown. This resulted in a markable enhancement of protein expression for RIG‐I and MDA5 as well as elevated phosphorylation levels of TBK1 and IRF3 in OC cells (Figure 7G,H). We also found IFN‐β levels were upregulated by RPS15AP12 KO or RPS15A knockdown (Figure S7T). These findings indicate that the RPS15AP12‐RPS15A axis targets innate immune signalling in OC cells.

## 4 DISCUSSION

Reversible regulation of ncRNAs by posttranscriptional m^6^A modification is crucial in cancer biology, with circRNAs, miRNAs, and lncRNAs being the most commonly modified ncRNAs.^33^ m^6^A modification enables circRNAs to regain translational capacity.^34^ The production of circRNAs is enhanced by METTL3‐mediated m^6^A, which stabilizes RNA transcripts and reduces RNA degradation.^35^, ^36^, ^37^, ^38^ Conversely, endoribonucleolytic breakage is observed in certain m^6^A‐modified circRNAs, leading to reduced RNA stability.^39^ Additionally, m^6^A modification facilitates circRNA back‐splicing and biosynthesis.^40^, ^41^, ^42^ For miRNAs, m^6^A primarily enhances the processing of pre‐microRNAs.^43^, ^44^, ^45^ Similar to circRNAs, m^6^A impacts the expression levels of lncRNAs by both promoting and inhibiting RNA degradation and stabilization.^46^, ^47^ Moreover, m^6^A modification increases the nuclear accumulation of lncRNAs.^48^ Genome‐wide landscape analysis has also identified m^6^A on pseudogene lncRNAs as frequently distributed in human cancers, including GBM and HNSCC, with significant correlations to the immunophenotype.^49^, ^50^ However, the intrinsic mechanisms of m^6^A regulation of pseudogenes remain underexplored. One study revealed that m^6^A enhances the RNA stability of pseudogene WTAPP1 and promotes WTAP translation in pancreatic cancer.^51^ In our study, m^6^A was frequently found on pseudogene‐derived lncRNAs, influencing their redistribution and activation in HGSOC. m^6^A plays a role in RNA surveillance by downregulating RNA stability, aided by the reader protein YTHDF2. These findings expand our understanding of m^6^A's surveillance of pseudogenes and its implications in cancer. Moreover, pseudogenes may regulate m^6^A deposition by directly or indirectly interacting with m^6^A regulators. DDX3X interacts with the m^6^A RNA demethylase ALKBH5, influencing the m^6^A modification of its target genes.^52^ AZGP1P2 binds to UBA1 and RBM15, acting as a “writer” of methyltransferase to form a complex, where RBM15 regulates the mRNA decay of TPM1 through m^6^A methylation.^53^ These observations illustrate a complex regulatory network between m^6^A and pseudogene‐lncRNAs.

In the present study, we found that the pseudogene RPS15AP12 functions as a lncRNA and competitively binds to RPS15A, acting as a miRNA sponge. The ceRNA hypothesis suggests that RNA transcripts containing miRNA‐response elements, mainly lncRNAs and circRNAs, can sequester miRNAs from other targets with the same MREs, thus regulating their expression.^54^ Sharing conserved miRNA seed target locations with PTEN, PTENP1 was the first found lncRNA generated from a pseudogene to target miR‐17, miR‐21, miR‐214, miR‐19, and miR‐26. lncRNAs are associated with the modulation of oncogenic and tumour‐suppressing pathways.^55^ Besides PTENP1, pseudogenes such as OCT4P4, BCAS5, CYP42P, and BRAFP1 have also been identified as capable of acting as ceRNAs. In OC, HMGA1P6, CTSLP8, and SDHAP1 are known to competitively bind to their paralogs, influencing tumour metastasis and paclitaxel resistance.^20^, ^21^, ^22^ Additionally, we observed positively correlated expression in most pairs, supporting ceRNA as a prevalent posttranscriptional regulatory mechanism. Most processed pseudogenes are located on different chromosomes from their paralogs, as they derive from reverse transcription. This trans regulation suggests that sequence homology and potentially secondary structure play more critical roles than chromosomal location in regulating gene expression.^56^ Therefore, the ceRNA regulation of their paralogs by pseudogenes should be considered a priority.

Aside from the ceRNA mechanism, lncRNA can bind to chromosomes in the nucleus to form RNA‐loop structures. TERRA, a lncRNA originating from telomere ends, associates with telomeres through an R‐loop‐dependent mechanism and is essential for the preservation of telomere integrity.^57^ lncRNA can influence histone modification and DNA methylation, thereby regulating gene expression. TARID, which activates TCF21 expression by inducing promoter DNA demethylation, is one such example.^58^ MAYEX is associated with the acetylated histone 4 lysine 16 (H4K16ac) epigenetic mark and can loop its locus to the entirety of the X chromosome, enhancing expression levels.^59^ lncRNAs can also bind to DNA and recruit proteins such as transcription factors (TFs). NR2F1‐AS1 interacts with NR2F1 mRNA and facilitates the recruitment of PTBP1, thereby enhancing the translation of NR2F1 and leading to the inhibition of ΔNp63 transcription. Furthermore, lncRNAs have the capacity to interact with RNA‐binding proteins (RBPs), influencing their localization, stability, and functional roles.^60^ OCC‐1 was observed to interact with HuR, facilitating HuR's association with the transducin repeat‐containing protein 1 (TrCP1) ubiquitin E3 ligase, thereby promoting its ubiquitination and subsequent degradation.^61^ The lncRNA EPB41L4A‐AS1 exhibits colocalization with histone deacetylase 2 (HDAC2) and Nucleophosmin 1 (NPM1) within the nucleolus. This interaction plays a crucial role in the transposition of HDAC2 from the nucleoplasm to the nucleolus, thereby influencing histone modification processes.^62^ In the present study, RPS15AP12 is distributed in both the nucleus and cytoplasm, predominantly in the cytoplasm. Therefore, RPS15AP12 could exhibit its role in the recruitment of TFs, regulation of histone modification, and DNA methylation. Moreover, aside from RPS15A, RPS15AP12 may target other molecules such as RBPs to form a complex regulatory network, which requires more investigations in further research.

Ribosomal protein S15A (RPS15A), a member of the ribosomal protein S family, is situated at the locus 16p12.3 on human chromosome 16 and encodes a highly conserved component of the 40S ribosomal subunit. RPS15A plays a crucial role in the interaction between capped mRNA and the small ribosomal subunit at the initial phases of translation.^23^ It has also been implicated in NF‐κB/ AKT, p53, and Wnt/β‐catenin pathways^23^, ^24^, ^25^ and is known to promote cell proliferation. In OC, transcriptional expression of RPS15A is inhibited by FOXN3, which suppresses cancer progression.^63^ By utilizing integrative RNA‐seq, we found that blocking the RPS15AP12/miR‐96‐3p/RPS15A axis had an anti‐tumour effect via innate immune responses, as it decreased OC cell proliferation, increased the expression of RNA sensors RIG‐I and MDA‐5, and activated the downstream type I IFN pathway. Virus infections normally trigger IFN signalling under normal physiological circumstances.^64^ Various pathogen‐associated molecular pattern (PAMP) sensors, including RIG‐I, MDA5, and TLR3, recognize abnormal viral RNA/DNA and activate downstream IFN pathways.^64^ Similarly, abnormal self‐derived RNA/DNA in tumours can activate IFN signalling through these sensors, inducing a “viral mimicry” state.^64^ Additionally, landscape analyses have explored pseudogene expression and immunophenotype using an immune score algorithm.^49^, ^50^ Mechanically, pseudogene lncRNA BRCA1P1 binds to the NF‐kB subunit RelA and inhibits its activation and the expression of ISGs.^9^ Possible binding of DDX3X by pseudogene RFPL1S‐202 to IFNB1 increases m^6^A modification, which in turn decreases p‐STAT1 and ISG expression.^52^ Emerging data suggests that immune cells displaying an immunosuppressive phenotype still possess anti‐tumour capabilities and that targeted interventions can successfully reprogram them to adopt an anti‐tumour phenotype.^65^ Targeting IFN signalling has proven effective in enhancing the anti‐PD‐L1 effect across various cancer types.^66^ In this study, we confirmed that RPS15AP12 competes with RPS15A for binding to miR‐96‐3p, thus inhibiting innate immune pathways. Our findings provide insights for enhancing immunotherapy effects in HGSOC, with further investigations required.

In summary, we identified frequent m^6^A modifications on pseudogene‐derived lncRNAs and their redistribution and activation in HGSOC in this study. m^6^A may play a role in RNA surveillance by reducing RNA stability. RPS15AP12‐lncRNA, regulated by m^6^A, competes with RPS15A for miR‐96‐3p binding, thereby inhibiting innate immune pathways and facilitating tumour progression. This indicates that RPS15AP12 could be a potential therapeutic target for OC.

## AUTHOR CONTRIBUTIONS

Tao Liu and Ping Yi conceptualized and supervised the research. Jie Xu and Yifei Ren were engaged in biostatistics and bioinformatics analysis. Jiayi Lu, Yifei Ren, Fengjiang Qin, Dan Yang, and Chunyan Tang performed most experiments. Yu Yang and Jing Xu helped with data analysis. Jie Xu drafted the manuscript. All authors read, reviewed, edited, and approved the final manuscript.

## CONFLICT OF INTEREST STATEMENT

The authors declare no conflict of interest.

## ETHICS STATEMENT

This study has been approved by the Ethics Committee of The Third Affiliated Hospital of Chongqing Medical University (Approval number: 2024‐011) according to the Chinese Ethical Regulations and conducted according to the guidelines of the Declaration of Helsinki.

## Supporting information



Supporting Information

## Data Availability

All datasets generated in this study have been deposited in the Sequence Read Archive (SRA) database under the accession number PRJNA1113826. All data are available from the corresponding author upon reasonable request.
