# Supplementary material for: Genome‐wide profiling of N6‐methyladenosine‐modified pseudogene‐derived long noncoding RNAs reveals the tumour‐promoting and innate immune‐restraining function of RPS15AP12 in ovarian cancer
Source: Clin Transl Med. 2025 Feb 25;15(3):e70249. doi: 10.1002/ctm2.70249 (PMC11859666; doi:10.1002/ctm2.70249)
Supplement: Supplementary file 1 — Supporting Information [file CTM2-15-e70249-s001.docx]

### Supplementary Information

**
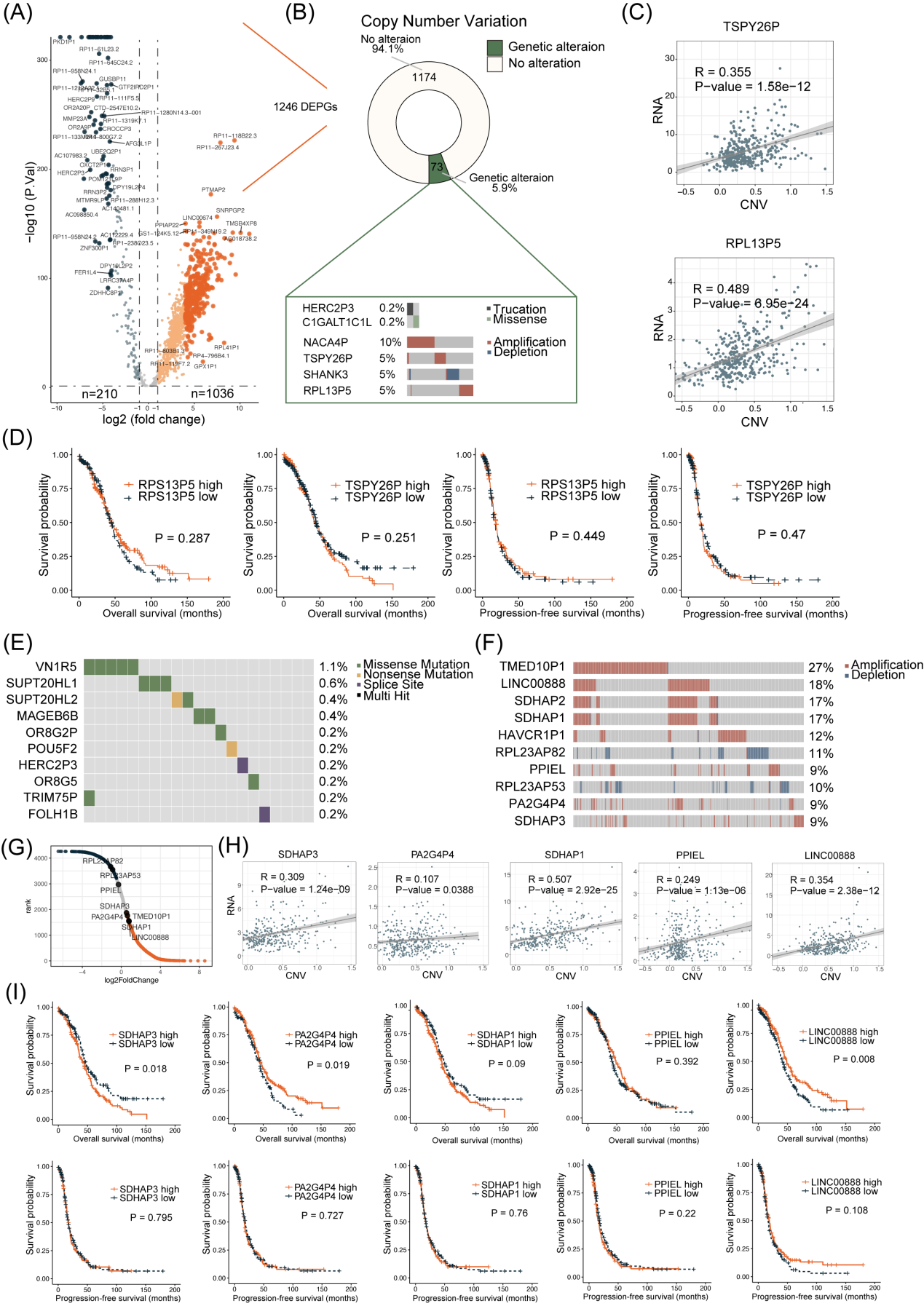
**

**Figure S1** (A) Volcano plot displaying 1246 DEPGs (limma R package with |Fold change| >1.5 and P-value < 0.05 ) of TCGA-OV (ovarian cancer) v.s. GTEx-ovary RNA-seq datasets. (B) Pie plot showing 73 pseudogenes out of 1246 DEPGs have genomic alterations. Pseudogenes with mutation and CNV frequencies >= 5 % according to cBioPortal database were displayed as representatives. (C) Correlation analysis between CNV and RNA levels of TSPY26P and RPL13P5 according to TCGA-OV data. (D) Kaplan-Meier OS and PFS analysis according to the expression of TSPY26P or RPL13P5. (E, F) Waterfalls plot showing representative mutation and CNV frequencies of pseudogenes. (G) The log2(fold change) and ranking of those pseudogenes with top genetic variation frequencies in TCGA-OV (Ovarian cancer) v.s. GTEx-ovary RNA-seq datasets. (H) Spearman’s correlation analysis between pseudogene RNA expression and the CNV level according to the TCGA-OV cohort. (I) Kaplan-Meier survival analysis of FAM86EP, BCASP2, RPL24P8 and RPS15AP12 expression according to the TCGA-OV cohort.

**
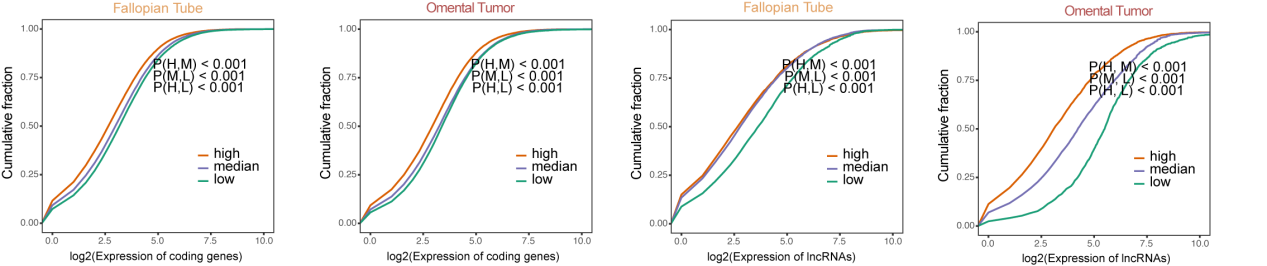
 Figure S2** CDF plot of mRNA and lncRNA expression in high, median and low m^6^A level groups. Wilcox analysis was applied in comparisons between multiple groups.


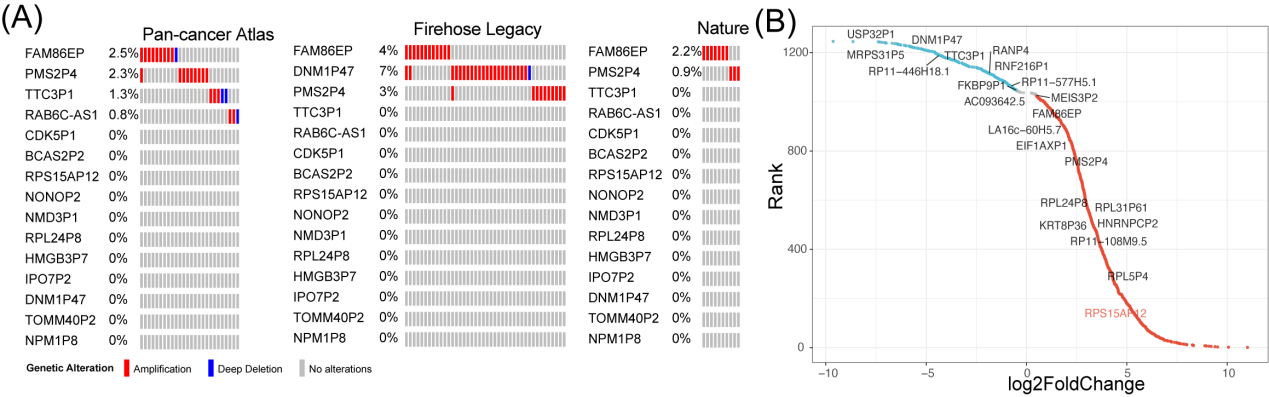


**Figure S3** (A) Waterfall plot of CNV frequencies of 15 differential-m^6^A pseudogenes in Pan-cancer Altas, Firehose Legacy and Nature datasets according to cBioPortal database. (B) The log2(fold change) and ranking of differential-m^6^A-modified pseudogenes in TCGA-OV (Ovarian cancer) v.s. GTEx-ovary RNA-seq datasets**.**


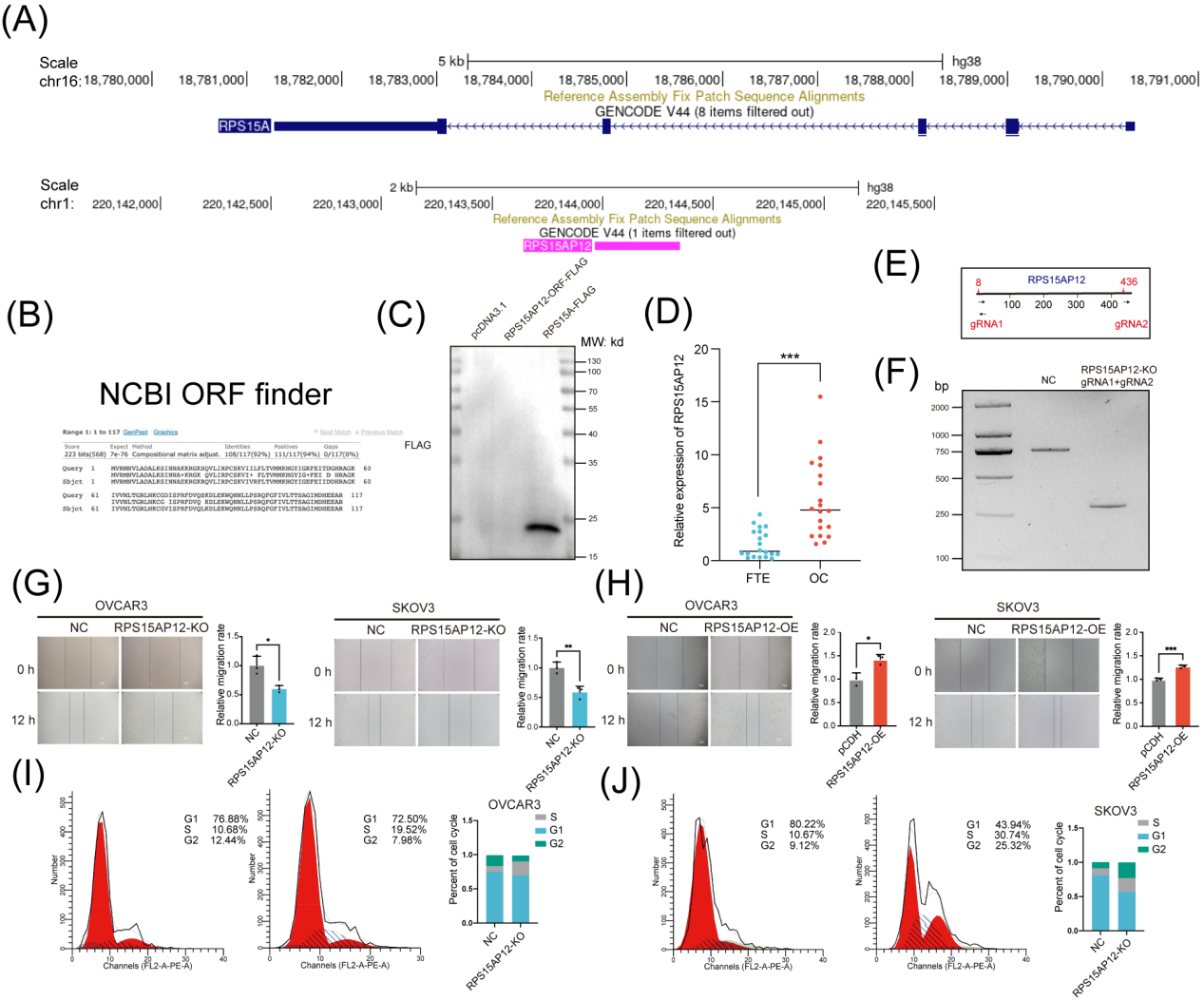


**Figure S4** (A) Gene structure of RPS15A and RPS15AP12 according to UCSC GENE BROWSER database. (B) Prediction for coding ability of RPS15AP12. (C) Western blot assays of cells with ectopic expression of FLAG-tagged ORF sequence of RPS15AP12. FLAG-tagged RPS15A was used as a positive control. (D) PCR assays of RPS15AP12 in clinical samples of OC tumor and normal fallopian tube epithelial (FTE) tissues. (E) Two guide RNAs (gRNAs) were designed for RPS15AP12 KO. (F) RPS15AP12 knockout in OC cells was confirmed by RT-PCR assays. The expected size of PCR amplicon from wild type (WT) is 759 bp (NC), whereas that from RPS15AP12 knockout (KO) is 300 bp (gRNA 1+2). (G, H) OVCAR3 and SKOV3 cells transfected with RPS15AP12 KO and RPS15AP12 OE were subjected to scratch wound-healing assays. The wound space was photographed at 0 h and 12 h. (I, J) Cell cycle was detected in OVCAR3 and SKOV3 cell lines upon RPS15AP12 knockdown.

**
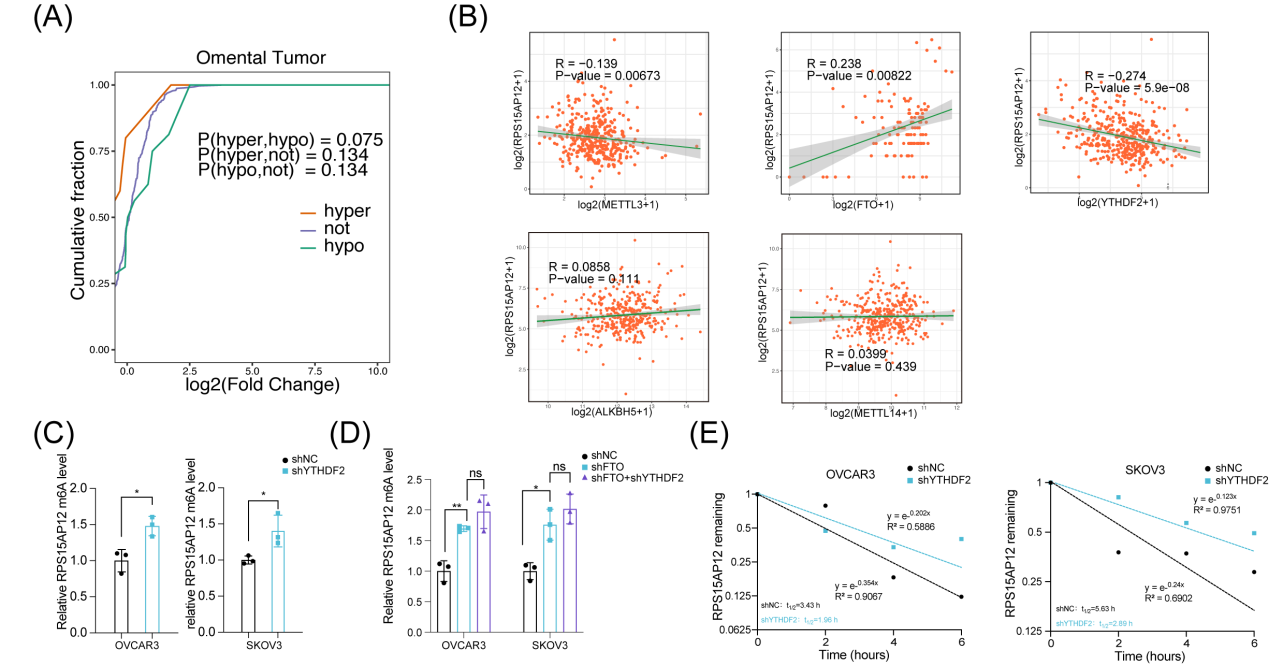
 Figure S5** (A) CDF plot of log2(fold change) of tumor v.s. normal in high, median and low m^6^A level group. Wilcox analysis is applied in comparisons between multiple groups. (B) Spearman’s correlation analysis between RPS15AP12 and m^6^A regulators’ RNA expression. (C) RT-qPCR assays detecting the expression of RPS15AP12-lncRNA upon YTHDF2 knockdown. (D) MeRIP-PCR detecting m^6^A level of RPS15AP12 upon FTO and YTHDF2 knockdown. (E) RNA half-life assays detecting the stability of RPS15AP12-lncRNA upon YTHDF2 knockdown.

**
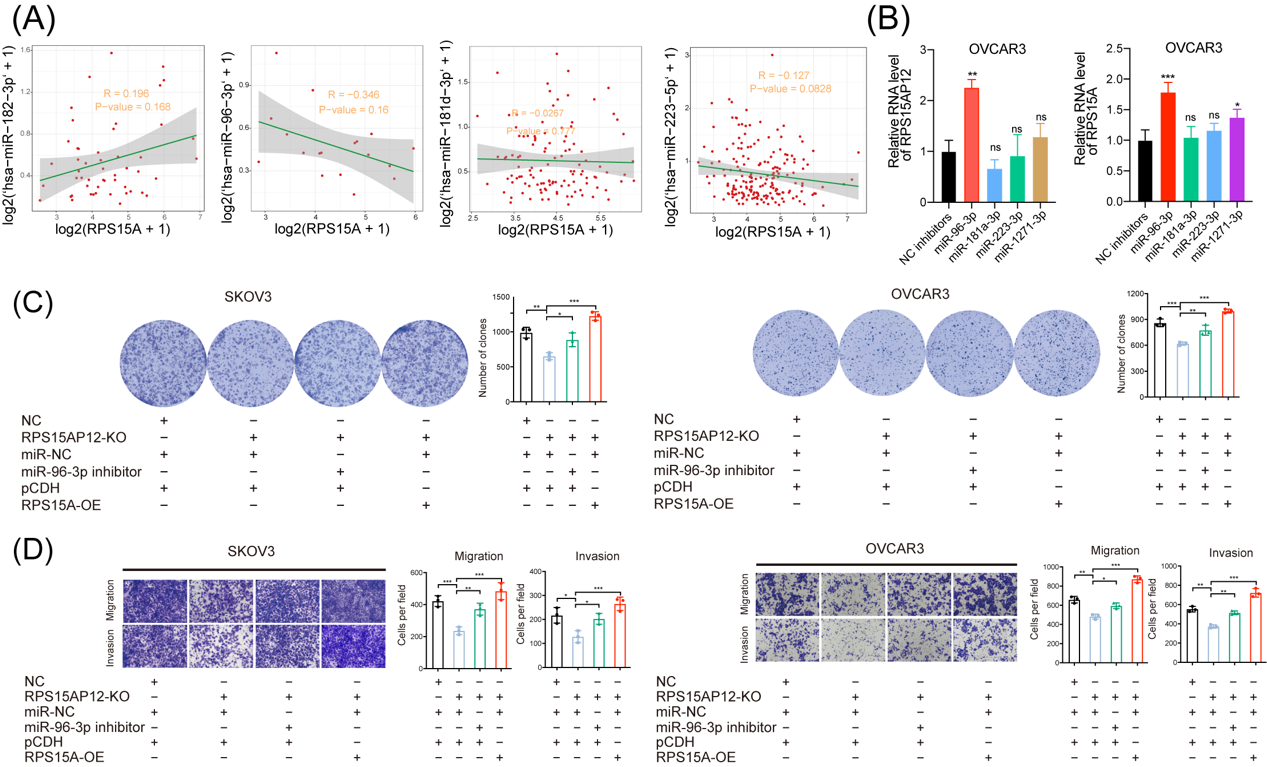
**

**Figure S6** (A) Spearman’s correlation analysis between miRNAs and RPS15A mRNA levels according to the TCGA ovarian cancer cohorts. (B) RT-qPCR detecting RPS15A mRNA expression upon miRNA inhibitors in ovarian cancer cells. (C) Colony formation assays were performed in control, RPS15AP12-KO and RPS15AP12-OE ovarian cells transfected with NC or miR-96-3p inhibitor. (D) Transwell assays detecting migration and invasion were performed in control, RPS15AP12-KO and RPS15AP12-OE ovarian cells transfected with NC or miR-96-3p inhibitor. One-way ANOVA, *P < 0.05, **P < 0.01, ***P < 0.001, NS, not significant.


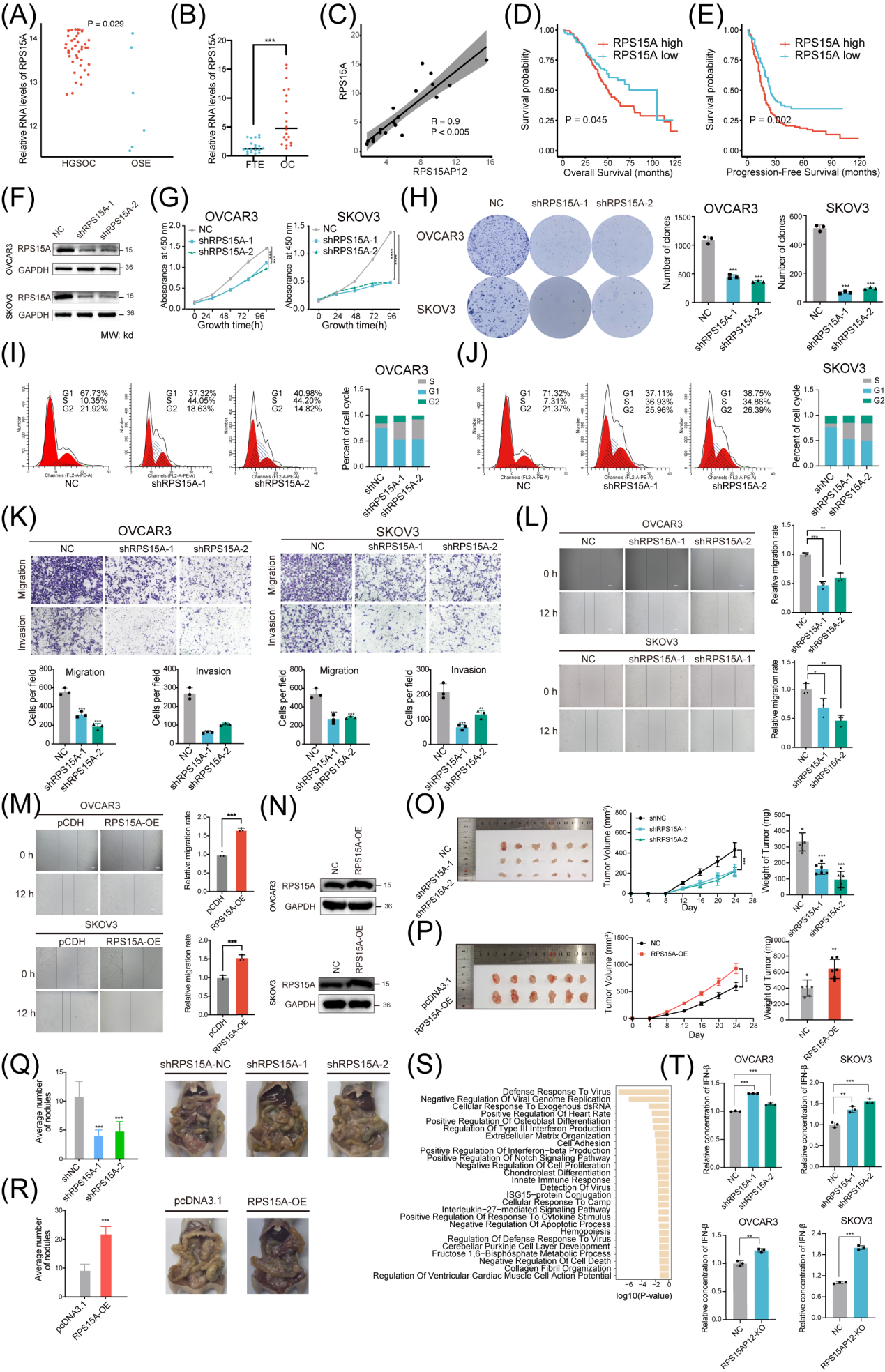


**Figure S7** (A) Boxplot of RPS15A expression in high grade serous ovarian cancer tumor and ovarian surface epithelial (OSE) samples in GSE27651 dataset. (B) RT-qPCR assays of RPS15A in clinical samples of OC tumor and normal fallopian tube epithelial tissues. (C) Spearman’s correlation analysis between RPS15AP12 and RPS15A expression in OC. (D, E) Kaplan-Meier survival analysis for OC patients with high RPS15A expression and low RPS15A expression according to the GSE32062 dataset. (F) Western blot detecting RPS15A protein levels upon knockdown of RPS15A. (G) CCK-8 assays of RPS15A knockdown in OVCAR3 and SKOV3 cell lines. (H) Colony formation assays performed in OVCAR3 and SKOV3 cell lines upon RPS15A knockdown. (I, J) Cell cycle assays detecting changes of OVCAR3 and SKOV3 cell lines upon RPS15A knockdown. (K, L)Transwell assays detecting migration and invasion of OVCAR3 and SKOV3 cell lines upon RPS15A knockdown. (M) OVCAR3 and SKOV3 cells with RPS15A knockdown or overexpression were subjected to scratch wound-healing assays. (N) Western blot detecting RPS15A protein levels upon overexpresion of RPS15A. (O, P) Effects of RPS15A knockdown and RPS15A overexpression on tumor weight and volume in the subcutaneous xenograft nude mouse model. (Q, R) The metastasis of OVCAR3 and SKOV3 cells with or without RPS15A knockdown and RPS15A overexpression to the peritoneal cavity of mice was assessed. (S) Functional enrichment analysis of 108 DEGs shared by RPS15A knockdown and RPS15AP12 knockout. (T) ELISA detecting the IFN-β level in OVCAR3 and SKOV3 cell lines upon RPS15AP12 knockout and RPS15A knockdown.
